# Health-Related Quality of Life in Adult Patients with Common Variable Immunodeficiency Disorders and Impact of Treatment

**DOI:** 10.1007/s10875-017-0404-8

**Published:** 2017-05-23

**Authors:** Nicholas L. Rider, Carleigh Kutac, Joud Hajjar, Chris Scalchunes, Filiz O. Seeborg, Marcia Boyle, Jordan S. Orange

**Affiliations:** 1 Texas Children’s Hospital and Baylor College of Medicine, 1102 Bates St, Houston, TX 77030 USA; 2 Section of Immunology, Allergy and Rheumatology, Texas Children’s Hospital, 1102 Bates St, Suite 330, Houston, TX 77030 USA; 3 Immune Deficiency Foundation, 110 West Road, Suite 300, Towson, MD 21204 USA

**Keywords:** Primary immunodeficiency diseases, common variable immunodeficiency, quality of life, reported health, physical health, mental health

## Abstract

**Purpose:**

Common variable immunodeficiency disorder (CVID) is a primary immunodeficiency disease (PIDD) often associated with severe and chronic infections. Patients commonly receive immunoglobulin (Ig) treatment to reduce the cycle of recurrent infection and improve physical functioning. However, how Ig treatment in CVID affects quality of life (QOL) has not been thoroughly evaluated. The purpose of a recent Immune Deficiency Foundation (IDF) mail survey was to assess the factors that are associated with QOL in patients with CVID receiving Ig treatment.

**Methods:**

A 75-question survey developed by the IDF and a 12-item Short Form Health Survey (SF-12) to assess QOL were mailed to adults with CVID. Mean SF-12 scores were compared between patients with CVID and the general US adult population normative sample.

**Results:**

Overall, 945 patients with CVID completed the surveys. More than half of the patients (54.9%) received intravenous Ig and 44.9% received subcutaneous Ig treatment. Patients with CVID had significantly lower SF-12 scores compared with the general US population regardless of sex or age (*p* < 0.05). Route of IgG replacement did not dramatically improve QOL. SF-12 scores were highest in patients with CVID who have well-controlled PIDD, lacked physical impairments, were not bothered by treatment, and received Ig infusions at home.

**Conclusion:**

These data provide insight into what factors are most associated with physical and mental health, which can serve to improve QOL in patients in this population. Improvements in QOL can result from early detection of disease, limiting digestive system disease, attention to fatigue, and implementation of an individual treatment plan for the patient.

**Electronic supplementary material:**

The online version of this article (doi:10.1007/s10875-017-0404-8) contains supplementary material, which is available to authorized users.

## Introduction

Primary immunodeficiency diseases (PIDD) comprises a group of over 300 disorders affecting immune system function. The most common, accounting for 35–63% of cases, is common variable immunodeficiency (CVID) [1–3], which is characterized by deficiencies in immunoglobulin (Ig) quantity and quality. Although the true incidence of PIDD is unknown, United States (US) estimates are between 1 in 1200 (35% CVID) [1] and 1 in 2000–2400 (20–28% B cell defects, i.e., CVID) [4] persons. Further, the average time from symptom onset to diagnosis ranges from 4.0 to 12.4 years [2, 5] (depending upon the method of the population surveyed); this delay in diagnosis may primarily reflect patients with CVID (accounting for 63% of the PIDD diagnoses in this survey) who present uniformly across the age continuum and are diagnosed at all ages [6]. The incidence of CVID is therefore likely underreported, with patients often misdiagnosed or undiagnosed [7, 8]. Increasing educational efforts and awareness have potentially resulted in increasing prevalence of the disorders [9].

Identifying CVID is challenging given varied and diverse presentations [7, 10]. While symptoms may occur in childhood, they typically manifest later in life [6, 11–13]. Affected patients are subject to recurrent, severe, or unusual infections. In some cases, patients develop autoimmune or gastrointestinal disorders and malignancies [12]. These frequent and severe events often cause missed work and school days, or even require hospitalization. Additionally, patients can have infectious or inflammatory complications that result in permanent impairments such as chronic lung disease and deficits in digestive and neurological function [12, 14–16]. For these and other reasons, patients with CVID report lower quality of life (QOL) compared to patients with other chronic diseases [17–19].

Early diagnosis is extremely important for improving patient QOL as delay in diagnosis can lead to further severe infections that can interfere with activities of daily living, and increase the risk for permanent impairments [20, 21]. These impairments are known to adversely affect patient-perceived health, a subjective global assessment of health which relates to QOL [22]. Impairments also confer a greater risk of depression and anxiety among patients with CVID [23]. Additionally, QOL is often overlooked and may be affected by various factors including physical limitations, patient perception of disease, access to care, and treatment options [22].

Patients with CVID often require lifelong treatment with Ig [24, 25], which may be administered intravenously or via the subcutaneous route. Immunoglobulin replacement is associated with improved QOL compared with no treatment [19]. Although all Ig formulations are effective in preventing infections [26, 27], some patients receiving intravenous Ig (IVIG) report “wear off” fatigue immediately prior to their next infusion [28]. These effects are reported less frequently in patients treated with subcutaneous Ig (SCIG) attributed to weekly small volume infusions and stable immunoglobulin G (IgG) serum levels [29].

For the above reasons, the Immune Deficiency Foundation (IDF) conducted a number of patient surveys to assess the health and well-being of patients diagnosed with PIDD [2, 14, 15, 30]. In the present study, a 75-item treatment survey (IDF survey) was developed and administered by the IDF along with the 12-item Short Form Health Survey (SF-12) to patients with CVID. Inclusion of the SF-12 was designed to evaluate QOL in patients with CVID in comparison to US mean scores as controls. The IDF survey was used to cross-reference responses from the SF-12 to particular patient characteristics of relevance to self-reported health outcomes in adult patients.

## Methods

### Survey Subjects

We conducted an in-depth health, wellness, and treatment survey of a portion of US-based patients contained in the IDF patient contact database. Individuals with the following criteria were identified via the IDF patient database: adult patients with PIDD, parent/caretakers of persons with PIDD, and persons from the IDF 2013 National Patient Survey identified as current users of Ig (*n* = 1083). An additional 2917 adult patients/caretakers identified in the IDF patient database as current users of Ig were randomly sampled from the IDF patient database giving our study a total sample size of 4000. The initial survey was mailed on December 20, 2013. A second mailing to nonrespondents was conducted on January 27, 2014. Data collection was completed on February 28, 2014. From this larger data set, 945 patients with CVID were evaluated for purposes of the current study.

### Survey Design and Administration

This was a two-part mail survey, comprising a 75-question survey (IDF survey; Fig. S1) and SF-12 (https://www.optum.com/optum-outcomes/what-we-do/health-surveys/sf-12v2-health-survey.html) designed for adults aged ≥18 years. The 10-item SF-10 (for children aged <18 years) was also included. Importantly, only adult patient responses were included in our analysis. The questionnaires were self-administered and anonymous.

### IDF Survey

Information collected from the IDF survey included issues related to diagnosis, Ig treatment, and health and QOL in the most recent 12 months. As this survey was only used for cross-referencing and categorization purposes, the full details will be reported elsewhere.

### SF-12v2 Your Health and Well-Being

The SF-12 is a patient-administered tool used to measure domains of patient physical and mental health [31, 32]. Items are scored on a scale of 1–100, with higher values indicating better health. Results from the SF-12 survey were used to compare health and QOL parameters in patients with PIDD from that of the general US population standardized to have a mean score of 50 ± 10.

The SF-12 comprises two component scores (physical component score [PCS] and mental component score [MCS]) and four subcategories/health domains for each component. Component scores are determined by the patient’s responses to the corresponding subcategories. Subcategories used to determine the PCS include physical functioning, role–physical, bodily pain, and general health. Subcategories used to determine the MCS include mental health, role–emotional, social functioning, and vitality.

### Statistical Analysis

Cohort partitioning and subgroup definitions were derived from IDF survey responses. Categories such as “control” and “impairment” were derived from patient-reported survey answers. Additional clinical features were also culled from the IDF survey responses and included age at diagnosis, type and route of Ig, bother of Ig therapy, and fatigue. IDF survey subgroup analyses were performed by comparing SF-12 responses.

Only adult patients (≥18 years of age) who were currently receiving Ig treatment for CVID and who had completed the SF-12 survey were included in the analyses. Descriptive analysis was carried out for all responses. For some response sets, the patient mean component scores (PCS and MCS) were compared with the US mean score of 50 (i.e., healthy population). Each variable with missing responses was evaluated to determine if the missing data made up a significant percentage (i.e., >30%) of the results. If the missing respondents made up a significant percentage of the responses, they were omitted from the evaluation. Two-sample *t* tests for unequal variances were performed to determine statistical significance between groups.

When comparing variables with more than two groups, such as the comparison between age groups, between levels of perceived of disease control, treatment locations, etc., two-sample *t* tests for unequal variances were performed between all possible combinations of responses to multiple response variables. These two-sample *t* tests compared the mean PCS and MCS scores of each response combination to determine if there was a significant difference (*p* < 0.05) in PCS and MCS scores between response levels of the variable.

Specific comparisons were made between SF-12 component scores of CVID patients and the US mean, and between SF-12 component scores of patients receiving SCIG treatment and patients receiving IVIG treatment.

#### Analysis of PCS and MCS

Two multivariate regression models were built to predict the PCS and MCS scores of patients using the variables from the IDF survey instrument. These univariate analysis was run to determine which variables included in the study had a significant impact on PCS and MCS scores.

The variables that were considered in the analysis included gender, treatment (SCIG vs IVIG), treatment location (at home, hospital outpatient, infusion suite, other), age, age (category), treating doctor (allergist or immunologist vs other), how well the treatment controls CVID (well to completely, adequately, less than or adequately to poorly), fatigue due to Ig therapy, overall bother when getting Ig therapy (not bothered at all, bothered a little bit, moderately bothered, or bothered quite a bit to extremely), patient health in the past 12 months (excellent, very good, good, fair, poor, or very poor), limitations in the past 12 months (no limitation, slight limitation, moderate limitation, or severe limitation), hospitalized overnight, hospitalized in ICU, inpatient/intensive care unit, inpatient operations in the past 12 months, outpatient operations in the past 12 months, permanent loss or impairments, digestive and/or lung impairments (vs no impairments), serious infections, severe side effects from Ig treatment, and delayed infusion (due to insurance).

Variables were considered to have a significant impact on PCS and MCS scores if there was a *p* value of <0.05 when the variable was included in a linear regression model with PCS and/or MCS, respectively. The coefficients for these variables were noted as the estimated difference in mean PCS or MCS score compared with the referent category of the variable.

## Results

### Results of the IDF Survey

#### Survey Response

Of the 4000 surveys mailed, 1608 were received. For the purposes of the present analysis, 476 respondents were initially excluded if they did not have a PIDD diagnosis, were not currently treated with Ig, were <18 years of age, did not fill out the SF-12 form, and/or mistakenly filled out the SF-10 form. Diagnoses of the remaining 1132 surveys included CVID (*n* = 945), IgG subclass deficiency (*n* = 75), agammaglobulinemia (*n* = 47), and “other” (*n* = 65). Due to the low number of diagnoses other than CVID, only survey data from the adult patients with CVID (*n* = 945) were considered for further analysis. Comparison data included a normative sample from the US general population (*N* = 4040; QualityMetrics 2009 database). Some questions were left unanswered, and therefore, the actual number of respondents included per question varied.

#### CVID Patient Demographics and Characteristics

Of the 945 patients with CVID in our cohort, the majority of patients were female (78%). All patients were ≥18 years of age with a median age of 52.9 (range 18–82) years. Time to diagnosis ranged from 0 to 68 years with a median age at diagnosis of 38.6 years. The majority of our cohort received a CVID diagnosis between ages 20 and 59 years (73.9%). Our study focused only upon patients receiving Ig replacement. From our cohort, 55% were receiving therapy by the intravenous route of administration (Table 1).

**Table 1:** Demographics and characteristics of adult patients with CVID (*N* = 945)

Characteristic	*n* (%)
Sex	
Male Female	208 (22.0%) 737 (78.0%)
Current age, years	
18–19 20–39 40–59 ≥60	8 (0.8%) 176 (18.6%) 417 (44.1%) 344 (36.4%)
Age at diagnosis, years^a^	
0–19 20–39 40–59 ≥60 Missing	143 (15.2%) 307 (32.6%) 388 (41.3%) 96 (10.2%) 8 (0.8%)
Treating physician	
Immunologist and allergist Other	822 (87.0%) 123 (13.0%)
Ig treatment type	
IVIG SCIG IMIG	519 (54.9%) 424 (44.9%) 2 (0.2%)
Location of Ig infusion^b^	
IVIG	
At home^c^ Infusion suite Hospital outpatient Other^d^ Missing	210 (40.5%) 147 (28.3%) 69 (13.3%) 69 (13.3%) 24 (4.6%)
SCIG	
At home^e^ Other^f^ Missing	413 (97.4%) 7 (1.7%) 4 (0.9%)
Ig administration type, by age group, years	
18–24	
IVIG SCIG	16 (36%) 43 (64%)
25–34	
IVIG SCIG	43 (52%) 39 (48%)
35–44	
IVIG SCIG	59 (48%) 64 (52%)
45–54	
IVIG SCIG	118 (54%) 100 (46%)
55–64	
IVIG SCIG	161 (59%) 111 (41%)
65–74	
IVIG SCIG	95 (59%) 66 (41%)
≥75	
IVIG SCIG	27 (63%) 16 (37%)

*CVID* common variable immunodeficiency, *Ig* immunoglobulin, *IMIG* intramuscular immunoglobulin, *IVIG* intravenous immunoglobulin, *SCIG* subcutaneous immunoglobulin

^a^Age at diagnosis was estimated through use of the IDF 75-question survey. Questions 4, 6a, and 6b relate specifically to this concern. IDF survey questions are located in Supplemental Fig. 1

^b^Patients treated with IMIG (*n* = 2) not included in the analysis

^c^Includes nurse infusion (*n* = 177) and self-infusion (*n* = 33 [infusion administered by: patient, *n* = 21; other family member, *n* = 8; nurse or other healthcare practitioner who was a family member, *n* = 2; missing response, *n* = 2])

^d^Includes doctor’s private office (*n* = 31), hospital clinic (*n* = 26), and other (*n* = 12)

^e^Includes self-infusion (*n* = 405) and nurse-administered infusion (*n* = 8)

^f^Includes infusion suite (*n* = 4), hospital outpatient (*n* = 1), hospital clinic (*n* = 1), and other (*n* = 1)

#### Ig Infusions

Following PIDD diagnosis and Ig treatment initiation, most patients (87%) reported being treated by an immunologist-allergist (Table 1). More than half of the patients (54.9%) received IVIG and 44.9% received SCIG treatment. Two patients (0.2%) received Ig via intramuscular infusion and were thus excluded from the analysis.

Nearly all SCIG (97.4%) and slightly less than half of IVIG (40.5%) infusions were administered at home (Table 1). The majority of patients treated with SCIG received their infusions once (80%) or twice (10%) weekly, and the remaining patients received treatment daily (2%), three times weekly (3%), or at lesser frequencies per month (5%). The majority of patients treated with IVIG (84%) received treatment either once every 3 (29%) or 4 (55%) weeks; the remaining patients received treatment 1–3 times per week (3.5%), every 2 weeks (7.6%), or ≥5 weeks (5%).

#### Patient-Reported Health and QOL

Slightly more than half of the patients (54.8%) felt their PIDD was well-controlled (Table S1). Control was defined by the patient’s response to item 42 on the IDF questionnaire (Fig. S1). Overall, most patients indicated that they were either “not bothered at all” or “bothered only a little bit” (76.99%) when they received their Ig treatment. However, patients were categorized by their survey responses as to whether they always (37.7%), occasionally (38.9%), or never (22.3%) had periods of fatigue or low energy between infusions. Compared with men, a significantly greater proportion of women noted that they always felt fatigue between Ig infusions. Other characteristics compared similarly between men and women. A greater percentage of patients treated with IVIG (46.0%) reported always experiencing periods of fatigue or low energy compared with patients using SCIG (28.5%); conversely, a greater percentage of patients treated with SCIG (30.9%) reported never experiencing fatigue or low energy compared with patients treated with IVIG (15.7%). The proportion of patients who reported occasionally experiencing fatigue or low energy was similar between those treated with SCIG (40.1%) or IVIG (38%).

Slightly more than half of the cohort rated their health as good to excellent (56.8%) and indicated that they had “none” or “slight” limitation in work, play, or normal physical activity due to their health in the past 12 months (52.8%) (Table S1). Within the past 12 months, the majority of patients had not been hospitalized overnight (76.5%), hospitalized in an intensive care unit (95.6%), or did not undergo an inpatient (87.7%) or outpatient (77.9%) surgical procedure (Table S1).

### Results of the SF-12 Survey and Comparison with a General US Adult Normative Population

#### Mean SF-12 Scores

**Fig. 1:**
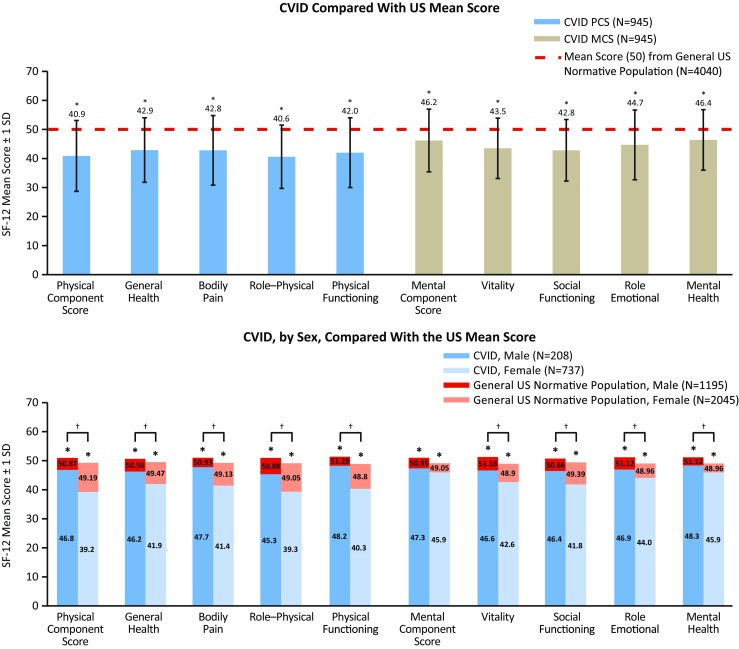
SF-12 mean physical (general health, bodily pain, role–physical, and physical functioning) and mental (vitality, social functioning, role–emotional, and mental health) component and domain scores for adult patients with CVID compared with the US mean score (*top*) and by sex compared with a general US normative population (*bottom*). ^*^ Significant difference between patients with CVID compared with the US mean score (*top figure*) and with the sex-specific general US normative population sample (*bottom figure*) (*p* < 0.05; lower than the US norm). ^†^ Significant difference between male patients with CVID compared with females with CVID (with the exception of MCS) (*p* < 0.05). *CVID* common variable immunodeficiency, *SD* standard deviation, *SF-12* 12-item Short Form Health Survey

**Fig. 2:**
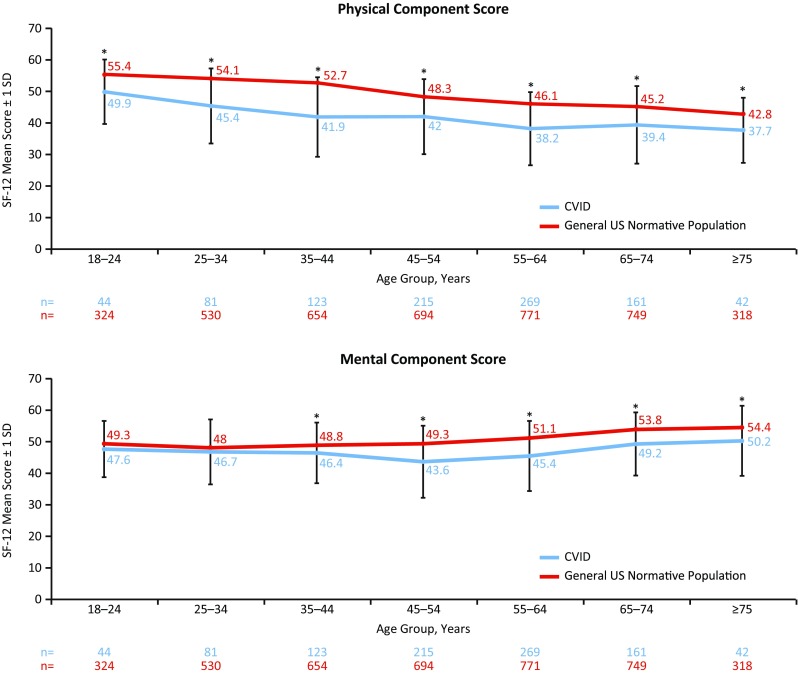
SF-12 mean physical and mental component scores for patients with CVID, by age group, compared with a general US normative population. ^*^ Significant difference between patients with CVID compared with the age-specific general US normative population (*p* < 0.05 for PCS and MCS; lower than the US norm). *CVID* common variable immunodeficiency, *SD* standard deviation, *SF-12* 12-item Short Form Health Survey

Mean SF-12 scores were compared between adult patients with CVID (*n* = 945) and the general US adult population normative sample (2009; *N* = 6045). Overall, patients with CVID had significantly diminished functional health and well-being, compared with the general US population (mean PCS score = 40.9 vs 50, *p* < 0.05; mean MCS score = 46.2 vs 50, *p* < 0.05) (Fig. 1). Patients had significantly lower mean SF-12 scores for PCS (Fig. 1) (*p* < 0.05) regardless of sex (Fig. 1) (*p* < 0.05). Patients also had significantly lower PCS and MCS compared with the US population regardless of current age (Fig. 2) and patient age at diagnosis (20–39 and 40–59 years age groups only) (*p* < 0.05). Patients who were diagnosed at ≥60 years of age scored significantly lower (*p* < 0.05) on the SF-12 for PCS, but not MCS compared with the US average (Fig. S2).

Although both males and females with CVID scored significantly lower on the SF-12 compared with the US average (*p* < 0.05, for both males and females), males scored significantly higher than females for PCS (*p* < 0.05) (Fig. 1). Similarly, between-age group analysis indicated that patients between 20 and 39 years of age had significantly higher (*p* < 0.05) SF-12 scores compared with patients aged 40–59 years for PCS and MCS and significantly higher (*p* < 0.05) scores for PCS and MCS when compared with patients ≥60 years. Patients aged 40–59 years also had significantly higher (*p* < 0.05) SF-12 scores for PCS but significantly lower scores for MCS compared with patients aged ≥60 years (Fig. S3).

#### Patient-Reported Health in the Past 12 Months

When considering their health in the past 12 months, patients who reported being in “fair-to-poor health” had significantly lower (*p* < 0.05) SF-12 scores for PCS and MCS compared with patients in “good-to-excellent health” (Fig. S4). However, compared with the general US population, both groups had significantly lower (*p* < 0.05) PCS and MCS scores.

#### Permanent Impairments

**Fig. 3:**
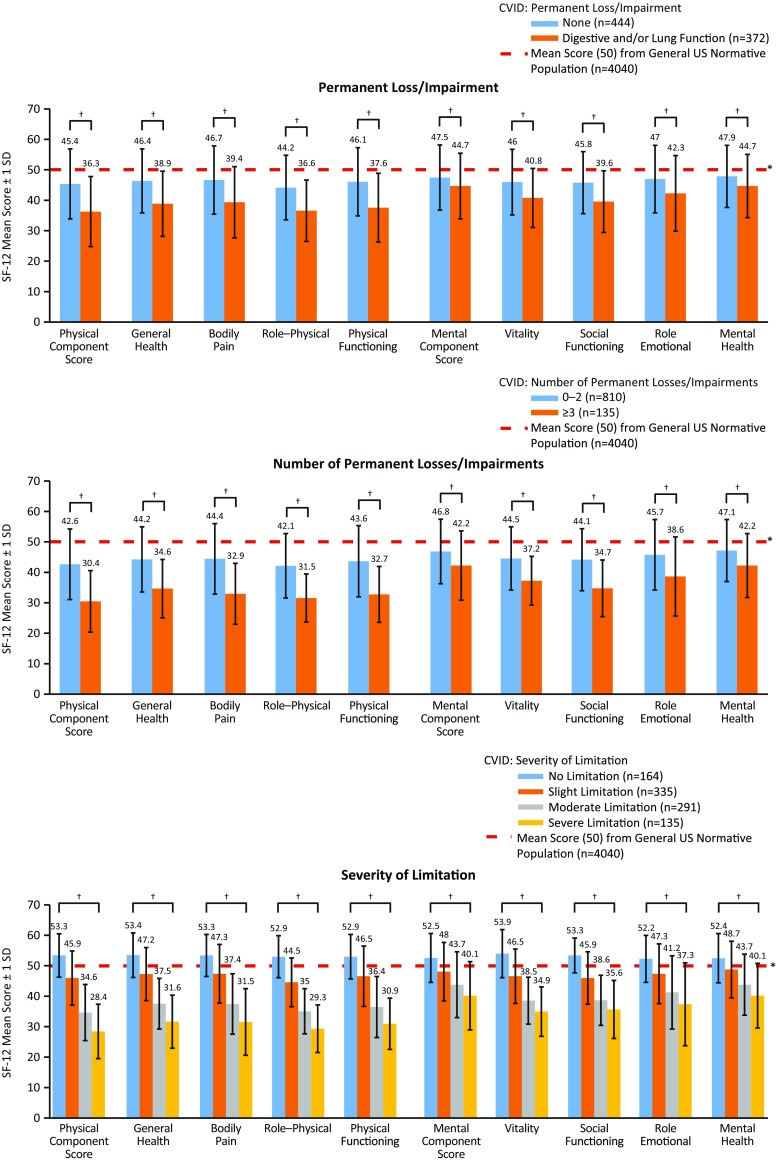
SF-12 mean physical (general health, bodily pain, role–physical, and physical functioning) and mental (vitality, social functioning, role–emotional, and mental health) component and domain scores for patients with CVID, by permanent loss/impairment (the number of permanent impairments refers to any type of impairment in the survey: digestive, kidney, liver, lung, or neurological function; hearing, mobility, vision, or others not listed), by number of permanent losses/impairments (between-group comparisons), and by severity of limitations in the past 12 months. Note, permanent impairment was patient described through the use of the 75 Question IDF Survey via item number 8. ^*^ Significant difference between all patients with CVID compared with the US mean score (*p* < 0.05; lower than the US norm) (*top* and *middle figures*) and significant difference between the mean SF-12 in the US normative population and patients with a “slight limitation” or worse (*p* < 0.05; lower than the US norm) and “no limitation” (*p* < 0.05; higher than the US norm) (all categories; *bottom figure*). ^†^ Significant difference between patients who had “no impairments” compared with patients who had a “permanent impairment” in digestive and/or lung function or between patients with 0–2 and ≥3 impairments (*p* < 0.05) (*top* and *middle figures*) and significant difference between patient groups (“no limitation” vs “slight limitation,” “slight limitation” vs “moderate limitation,” and “moderate limitation” vs “severe limitation”) (*p* < 0.05) (*bottom figure*). *CVID* common variable immunodeficiency, *SD* standard deviation, *SF-12* 12-item Short Form Health Survey

Overall, patients scored significantly lower (*p* < 0.05) on the SF-12 for MCS and PCS compared with the US general population, regardless of having or not having a permanent impairment in digestive and/or lung function (Fig. 3 [top]). Between-group comparisons revealed that patients who had no such impairments had significantly higher (*p* < 0.05) scores overall, compared with those with permanent functional loss. When patients were evaluated by number of any type of permanent impairment (i.e., digestive, kidney, liver, neurological, and lung function; hearing; mobility, vision, or “other”), those indicating ≥3 impairments scored significantly lower (*p* < 0.05) for PCS and MCS, compared with patients with 0–2 impairments (Fig. 3 [middle]). Importantly, patients who report digestive impairments (with and without lung impairments) tend to have a significantly lower PCS and MCS scores than other CVID patients.

#### Severity of Limitations in the Past 12 Months

Patients reporting any degree of physical limitation due to PIDD in the past 12 months (“slight limitation” or worse) had significantly lower (*p* < 0.05) SF-12 scores for PCS and MCS compared with the general US population, with scores numerically decreasing with increased severity (Fig. 3 [bottom]). In contrast, patients who reported having “no limitation” scored significantly higher than the US general population for PCS and MCS (*p* < 0.05).

Between-group analysis showed that patients who reported having “no” limitations scored significantly higher (*p* < 0.05) on the SF-12 for PCS and MCS compared with patients who reported “slight” limitations. Similarly, patients who had “slight” limitations and patients who had “moderate” limitations scored significantly higher (*p* < 0.05) on the SF-12 for PCS and MCS compared with patients who reported “moderate” or “severe” limitations.

#### SCIG vs IVIG: Comparison of Ig Administration Route

**Fig. 4:**
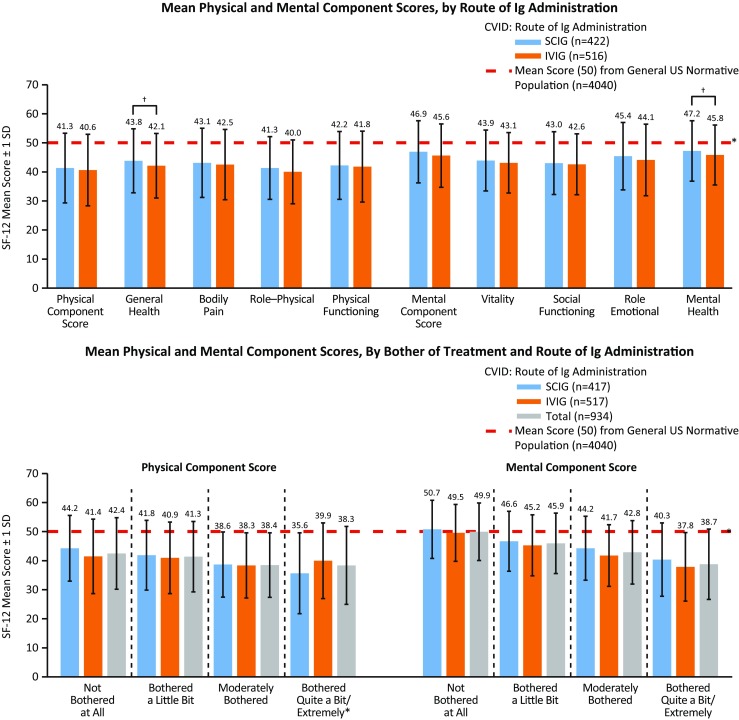
SF-12 mean physical (general health, bodily pain, role–physical, and physical functioning) and mental (vitality, social functioning, role–emotional, and mental health) component scores for patients with CVID, by route of Ig administration (*top*) and by bother of treatment and route of Ig administration (There were significant between-group differences between “not at all bothered” vs “bothered a little bit” for MCS (*p* < 0.05); “not at all bothered” vs “moderately bothered” for PCS and MCS (*p* < 0.05); and “not at all bothered” vs “extremely bothered” for PCS and MCS (*p* < 0.05). There were no significant differences by type of Ig administration (SCIG vs IVIG)) (*bottom*), compared with a US normative population. ^*^ Significant difference between any route of Ig administration (SCIG or IVIG) compared with a US normative population (*p* < 0.05; lower than the US norm; *top figure*) and significant difference in mean SF-12 scores between a general US normative population and patients who were “moderately bothered” or worse (PCS and MCS) and “not at all bothered” (PCS) (*p* < 0.05; lower than the US norm; *bottom figure*). ^†^ Significant difference between SCIG and IVIG (*p* < 0.05). *CVID* common variable immunodeficiency, *Ig* immunoglobulin, *IVIG* intravenous administration of Ig, *SCIG* subcutaneous administration of Ig, *SD* standard deviation, *SF-12* 12-item Short Form Health Survey

Regardless of route of Ig administration (SCIG or IVIG), patients with CVID had significantly lower (*p* < 0.05) SF-12 score for PCS and MCS compared with the general US population (Fig. 4 and Table S2). When stratified by age, the proportion of patients treated with SCIG vs IVIG was higher among younger (aged 18–24 years) patients; IVIG was used more frequently in all other (older) age groups (Table 1). No other differences were found between age groups.

#### Perception of Control of PIDD via Ig Treatment Administration Route

Higher SF-12 scores for PCS and MCS were associated with improved PIDD control. Between-group comparisons showed that patients who perceived to have well-controlled PIDD scored significantly higher for PCS and MCS compared with patients perceived to have adequately controlled (*p* < 0.05) and less than adequately/poorly controlled (*p* < 0.05) PIDD. Similarly, patients who perceived to have adequately controlled PIDD scored significantly higher for PCS and MCS, compared with patients who perceived to have less than adequately/poorly controlled PIDD (*p* < 0.05) (Figs. S5 and S6 and Table S2). When stratified by route of Ig administration, MCS was significantly higher (*p* < 0.05) in patients treated with SCIG compared with IVIG in those perceived to have “less than adequately/poorly controlled” PIDD (Fig. S5).

#### Periods of Fatigue or Low Energy Between Ig Treatments

**Fig. 5:**
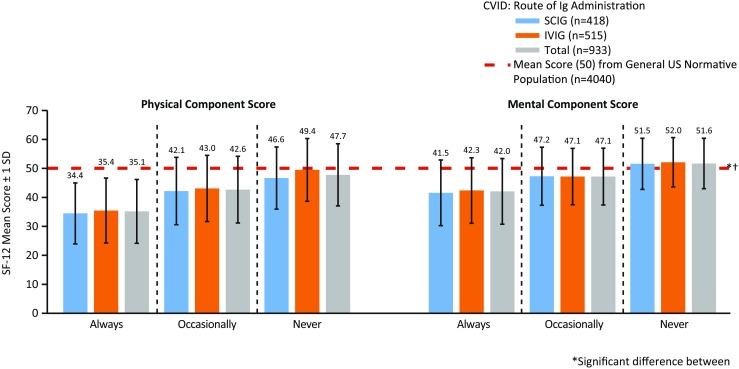
SF-12 mean physical (general health, bodily pain, role–physical, and physical functioning) and mental (vitality, social functioning, role–emotional, and mental health) component scores for patients with CVID, by route of Ig administration (There were no significant differences in mean SF-12 scores between SCIG or IVIG; between-group comparisons indicated a significant difference in SF-12 scores between patients who reported “never” experiencing postinfusion fatigue and patients who reported “always” or “occasionally” experiencing postinfusion fatigue (*p* < 0.05)) (“Does the patient experience periods of fatigue or low energy between Ig treatments?”). ^*^ Significant difference between patients who “never” experience periods of fatigue or low energy compared with the general US population normative sample (*p* < 0.05) (lower PCS than the US norm and higher MCS than the US norm). ^†^ Significant difference between patients who “occasionally” and “always” experience periods of fatigue or low energy compared with a US normative population (*p* < 0.05) (lower PCS and MCS). *CVID* common variable immunodeficiency, *Ig* immunoglobulin, *IVIG* intravenous administration of Ig, *MCS* mental component score, *PCS* physical component score, *SCIG* subcutaneous administration of Ig, *SD* standard deviation, *SF-12* 12-item Short Form Health Survey

Patients with CVID who indicated having “never” experienced fatigue or low energy between Ig infusions had significantly lower mean SF-12 scores compared with the general US population for PCS (Figs. 5 and S7 and Table S2). Interestingly, these patients scored higher (*p* < 0.05) for MCS compared with the US average. Patients reporting occasionally experiencing periods of fatigue or low energy between had significantly lower (*p* < 0.05) SF-12 scores for MCS and PCS.

Between-group comparisons indicated a significant difference in SF-12 scores between patients who reported “never” experiencing postinfusion fatigue and patients who reported “always” or “occasionally” experiencing postinfusion fatigue (*p* < 0.05; Figs. 5 and S7, Table S2). When stratified by experience of fatigue or low energy between treatments and route of Ig administration, there were no significant differences in mean PCS and MCS scores between patients receiving SCIG treatment and patients receiving IVIG treatments (Fig. 5).

#### Location of Ig Treatment Administration

**Fig. 6:**
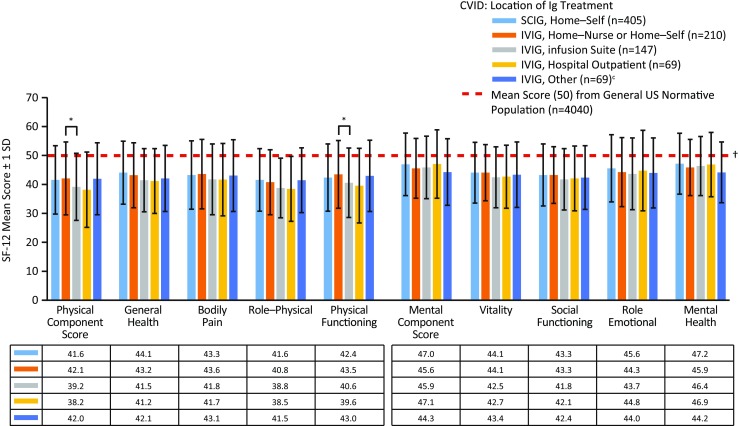
SF-12 mean physical (general health, bodily pain, role–physical, and physical functioning) and mental (vitality, social functioning, role–emotional, and mental health) component and domain scores for patients with CVID, by Ig treatment location (includes doctor’s private office (*n* = 31), hospital clinic (*n* = 26), and other (*n* = 12)) (“Where does the patient usually receive Ig therapy?”). ^*^ Significant difference between patients who received IVIG at home vs at an infusion suite (*p* < 0.05). ^†^ Significant difference between all patients with CVID compared with the US mean score (*p* < 0.05; lower than the US norm). *CVID* common variable immunodeficiency, *Ig* immunoglobulin, *IVIG* intravenous administration of Ig, *SCIG* subcutaneous administration of Ig, *SD* standard deviation, *SF-12* 12-item Short Form Health Survey

The majority of patients received IVIG “at home, nurse infused” (40.5%) or at an “infusion suite” (28.3%); patients who received home infusions had higher PCS and physical functioning scores compared with patients who received treatment at an infusion suite (Fig. 6 and Table 1). As the vast majority of patients received SCIG treatment at home (97.4%), between-location comparisons were not statistically feasible.

#### Bother of Ig Treatment

Patients scored significantly lower (*p* < 0.05) on the SF-12 for PCS and MCS compared with the general US population, regardless of how much they were bothered by their Ig treatment (“moderately bothered” or worse) (Figs. 4 and S8 and Table S2). Patients who were “not at all bothered” also scored significantly lower (*p* < 0.05) for PCS compared with the general US population.

However, between-group comparisons showed that patients who were “not at all bothered” scored significantly higher (*p* < 0.05) on the SF-12 for MCS compared with patients who were “bothered a little bit” (Figs. 4 and S8 and Table S2). Further, patients who were “not at all bothered” scored significantly higher (*p* < 0.05) for PCS and MCS on the SF-12 compared with patients who were “moderately bothered,” and significantly higher for PCS and MCS, compared with patients who were “extremely bothered” by treatment. When comparing any level of bother by route of Ig administration, there were no significant differences in mean SF-12 scores for PCS and MCS (Fig. 4).

#### Significant Variables Affecting PCS and MCS Scores Within the Cohort—Univariate Analysis

Perceived good health status over the preceding 12 months, lack of fatigue, and younger age positively affected the PCS to the greatest extent (Fig. S9 and Table S3), whereas very poor health status and limitations impacted lower PCS the most. Values for the MCS were also improved in patients without fatigue who had excellent perceived health (Fig. S10 and Table S4). Interestingly, advanced age generally favored a higher MCS which was in contrast to the findings noted for the PCS. Lower MCS values were influenced markedly by extreme bother with Ig therapy as well as severe functional limitations.

## Discussion

The purpose of this survey was to evaluate health status in patients with CVID and ascertain disease-specific factors of greatest importance for mental and physical well-being. Here, we report the single largest cohort focusing solely on QOL in patients with CVID. Overall, 945 patients with CVID who completed the SF-12 survey and indicated they were currently treated with Ig were included in the analysis. This is similar to the number of respondents to a 2008 patient treatment survey by the IDF [15] in which 1030 patients with PIDD who were treated with Ig were assessed. All responses were compared with the general US population in order to contextualize PIDD—something that has not previously been performed to this extent.

The majority of patients in our survey were diagnosed with CVID as adults and had some form of permanent impairment and/or loss. This is supported by data indicating that patients with CVID are diagnosed a mean age of 20–43 years [6, 33, 34] and have several impairments, with chronic lung disease being the most common [4]. Data from the current analysis also indicated that, overall, adult patients with CVID had significantly diminished mental and physical well-being compared with the general US population (Fig. 1). These findings are in contrast to data from a smaller survey in which patients with CVID (*N* = 16) scored close to the US average on the 36-item Short Form Health Survey (SF-36), which is a 36-item health item used to assess physical and mental health [36]. We noted significantly lower SF-12 scores in females for all metrics except the MCS (Fig. 1). This may reflect the fact that the majority of our cohort is female (78%; Table 1) or that there are important female-specific health impacts conferred by CVID as suggested in previous studies [35]. Additionally, when we investigated QOL metrics by age, our findings suggested that while lower than the general population, PCS and MCS in patients with CVID generally paralleled the normative trend (Fig. 2). Interestingly, PCS values generally dropped with age, but the MCS showed a trend toward higher values in the patients aged 75 and older (Fig. S9, Table S3, Fig. S10, and Table S4). This may reflect a degree of coping related to disease management. A similar phenomenon has been noted previously in patients with chronic disease in general (Figs. S9 and S10) [36, 37].

We noted expected reductions in QOL in all metrics for patients with impaired organ function and permanent disability (Fig. 3). Similarly, severity of limitation inversely correlated with QOL (Fig. 3). These results speak to the marked psychosocial effects conferred by CVID and chronic disease in general and are in line with other reports of disease impact upon QOL in CVID patients [38]. They may also relate to the difficulty in optimally managing all features of this disorder. We find that QOL impairments decline in lockstep with increased functional limitation, fatigue, and organ impairment. This further underscores the need for early diagnosis, limitation of organ disease, evaluating and treating fatigue, and intentional maintenance of daily function in ongoing management of CVID. These findings also are in concert with our earlier conclusions regarding patient-perceived health that emphasized many of these same messages.

Patients who reported no limitations in work, play, or normal physical activity as a result of their health in the past 12 months had higher SF-12 scores overall compared with the US mean. One possible explanation is that patients with CVID may have greater awareness of their physical health compared with the average (i.e., healthy) person. Previous reports from our group have observed the importance of physical activity and perceived health [22]. Self-perception of mental and physical health has been shown to be reliable indicators of QOL, more so than objective measures, such as wealth or social status [39].

We also sought to understand how Ig replacement therapy affected QOL. In general, IVIG and SCIG were tolerated similarly. We noted a modestly improved SF-12 Mental Health score for patients on SCIG replacement over IVIG (Fig. 4; *p* < 0.05) and improved MCS for those receiving SCIG vs IVIG who rated themselves as less than adequately controlled disease (Fig. S6).There may be some benefit from a mental health perspective when the patient is in control of medication administration as is the case with SCIG replacement. These findings support another study where patients were self-selected to continue treatment with SCIG 20% following a 12-month phase 3 clinical trial and had a favorable experience with SCIG treatment as noted by higher QOL scores [40]. However, a direct effect of route of therapy upon QOL is not ascertainable as other factors related to choice of therapy may supervene and contribute. Importantly, patient-specific features related to treatment route selection may influence subsequent QOL more than the route itself.

Our cohort also noted no difference in fatigue or “low energy” conferred by route of therapy; however, there was a direct correlation for SF-12 scores and degree of fatigue (Fig. 5) which is not surprising. Similarly, minor improvements in PCS were found in patients receiving IVIG at home compared with those receiving IVIG in an infusion suite (Fig. 6). Bother of treatment was also independent of Ig administration route in this cohort (Fig. 4). These findings suggest that the method of Ig replacement (intravenous or subcutaneous) is not as important as the burden of disease in its impact upon QOL among patients with CVID. More importantly, given the residual differences from the US population in QOL despite any of these interventions, our analysis suggests that there is still ample room for improvement with regard to the well-being of PIDD patients.

### Limitations

While the SF-12 accurately reflects MCS and PCS, it lacks the precision of the SF-36 [41]. Both the SF-12 and SF-36 compare a sample population with a general US population; comparisons are made via standardized scoring of the survey in relation to the mean US score. The SF-36, which was used to develop the SF-12, also measures the same eight subcategories to calculate MCS and PCS. However, the subcategories should not be used for direct comparison of groups; thus, we have only used the PCS and MCS for statistical comparison and report the results of the subcategory data for reader interest and completeness only (Figs. 1, 3, 4, 6, S6, S7, and S8). The SF-12 was appropriate for the current analysis as it accompanied a much longer survey. It can also be completed more rapidly than the SF-36 (approximately 2 vs 8 min, respectively) [32] or the PROMIS-29 (approximately 20 min) [42], which is another patient-reported survey that assesses physical, mental, and social health based on the following subcategories: physical function, anxiety, depression, fatigue, sleep disturbance, satisfaction with social role, pain interference, and pain intensity [43]. Shorter surveys may reduce respondent burden and increase the likelihood that the survey is completed and returned. The IDF is currently administering the PROMIS-29 to patients with PIDDs. However, the SF-12 and SF-36 have been previously used extensively in evaluating health-related QOL in this population [18, 19, 23, 44, 45], whereas there is little published research on the validity of the PROMIS-29 for patients with PIDD at this time. These three scales are general measures of health-related QOL and not meant for a specific clinical population. Most recently, a 22-item QOL survey specifically for patients with PIDD has been developed and is being validated from interim results from 76 patients (NCT02542228) [46]. This has great potential to identify distinct factors associated with QOL in the PIDD population.

There is also the possibility of recall bias, since patients were asked to report on their health in the past 12 months. The SF-12, however, is considered a reliable and valid measure across populations [47–49]. As with any survey, outcomes were subject to nonresponse bias. However, the number of responders to this survey was similar to previous IDF surveys and deemed to have adequate power. It should be noted that all data contained in this manuscript is derived solely from patient responses in light of the focus upon QOL and patient-reported health.

Correlation with the MCS and PCS, and not subscale data, was performed to show relevance. While not specifically validated, the IDF questionnaire was developed by immunologists, experienced nurses, and patients. Many of the questions included have been in use by the IDF for over 30 years and have yielded similar results on other populations.

Additionally, selection bias represents another limitation of this study. This survey was not a random probability sample as the data was collected from individuals who are registered with the IDF. These patients may be more engaged in their treatment of CVID compared to patients who are not registered with the IDF. We cannot generalize our results to all patients with CVID as there may be inherent difference between these two groups of patients. These differences, however, are unknown and beyond the scope of the current study.

### Conclusion

Although CVID is most associated with deficits in physical health, our study also found that CVID affects broader well-being. These data provide insight into what factors are most associated with physical and mental health, which can serve to improve QOL in patients in this population. Importantly, this analysis associated the following factors with higher QOL scores in CVID: younger age, male sex, early diagnosis, less functional impairment, less/lack of organ-associated disease, no postinfusion fatigue, and use of SCIG or IVIG replacement in the home setting. We suggest that improved QOL relies on early detection of disease and implementation of an individual treatment plan for the patient. Future evaluation of QOL in CVID, or perhaps primary immunodeficiency in general, should focus on metrics that are disease specific such as organ and functional impairments. Also, identifying why women report significantly diminished QOL would be of great value.

## Electronic Supplementary Material

Fig S1 (PDF 13734 kb).

Fig S2 (JPEG 26 kb).

High resolution image (EPS 928 kb).

Fig S3 (JPEG 25 kb).

High resolution image (EPS 900 kb).

Fig S4 (JPEG 24 kb).

High resolution image (EPS 887 kb).

Fig S5 (JPEG 41 kb).

High resolution image (EPS 924 kb).

Fig S6 (JPEG 41 kb).

High resolution image (EPS 916 kb).

Fig S7 (JPEG 40 kb).

High resolution image (EPS 878 kb).

Fig S8 (JPEG 47 kb).

High resolution image (EPS 964 kb).

Fig S9 (PDF 108 kb).

Fig S10 (PDF 109 kb).

ESM 1 (DOCX 200 kb).

Table S1 (DOCX 51 kb).

Table S2 (DOCX 54 kb).

Table S3 (DOCX 47 kb).

Table S4 (DOCX 43 kb).
