# Supplementary material for: Health-Related Quality of Life in Adult Patients with Common Variable Immunodeficiency Disorders and Impact of Treatment
Source: J Clin Immunol. 2017 May 23;37(5):461–75. doi: 10.1007/s10875-017-0404-8 (PMC5489588; doi:10.1007/s10875-017-0404-8)
Supplement: Supplementary file 1 — (PDF 13734 kb). [file 10875_2017_404_MOESM1_ESM.pdf]

Immune Deficiency Foundation

INSTRUCTIONS

IDF 2013 Treatment Survey

- **Adult patients:** Follow Instructions A
- **Adult patients with PI with a child with PI:** Follow Instructions B
- **Parent of a child with PI:** Follow Instructions C

FOR ADULT PATIENTS - INSTRUCTIONS A

1. Complete the IDF Treatment Survey.

2. Complete the survey *SF-12 Your Health and Well-Being*.

3. Place both the completed SF-12 and the completed IDF Treatment Survey into the return envelope provided for you and drop it in the mail. There is no need to place any postage on the return envelope. There is no need to return the uncompleted SF-10.

FOR ADULT PATIENTS WITH PI WITH A CHILD WITH PI - INSTRUCTIONS B

1. Complete the IDF Treatment Survey.

2. Complete the survey called *SF-12 Your Health and Well-Being*. IDF is only asking questions about YOUR health not of your child or children.

3. Please place both the completed SF-12 and the completed IDF Treatment Survey into the return envelope provided for you and drop it in the mail. There is no need to place any postage on the return envelope. There is no need to return the uncompleted SF-10.

FOR THE PARENT OF A CHILD WITH PI - INSTRUCTIONS C

1. Complete the IDF Treatment Survey.

2. Complete the survey called *SF-10 Health Survey for Children* **only** if your child is between the ages of 5 and 18. If your child is under the age of 5, you only need to complete and return the IDF Treatment Survey.

3. Place both the completed SF-10 and the completed IDF Treatment Survey into the return envelope provided for you and drop it in the mail. There is no need to place any postage on the return envelope. There is no need to return the uncompleted SF-12.

Please turn the page and begin the IDF 2013 Treatment Survey

Immune Deficiency Foundation

TREATMENT EXPERIENCES AND PREFERENCES AMONG PATIENTS WITH PRIMARY IMMUNODEFICIENCY: 2013

1. Are you a **patient** with a primary immunodeficiency disease (PI) or **parent/caregiver** of a child **in the household** with PI?

- ☐1 PI patient → **CONTINUE**
- ☐2 Parent/caregiver → **CONTINUE**
- ☐3 Both → **CONTINUE**
- ☐4 Neither → **PLEASE STOP**

IF YOU ARE A PATIENT WITH PI, PLEASE ANSWER SURVEY QUESTIONS ABOUT YOURSELF. IF YOU ARE NOT A PATIENT, PLEASE ANSWER SURVEY QUESTIONS ABOUT THE OLDEST CHILD WITH PI IN HOUSEHOLD.

2. What is the date of birth of the (adult patient/oldest child) in the household with a primary immunodeficiency disease?

______ MONTH ______ DAY ______ YEAR

3. What is the gender of that person?

- ☐1 Male
- ☐2 Female

4. At what age (in years) was that person first diagnosed with a primary immunodeficiency disease?

______ Age at diagnosis
☐00 Less than one year old

5. What is the specific diagnosis of that person's immunodeficiency disease?

- ☐1 Agammaglobulinemia (XLA)
- ☐2 Ataxia Telangiectasia
- ☐3 Common Variable Immunodeficiency (hypogammaglobulinemia)
- ☐4 Hyper IgM Syndrome
- ☐5 IgA Deficiency
- ☐6 IgG Subclass Deficiency
- ☐7 Severe Combined Immune Deficiency
- ☐8 Specific Antibody Deficiency
- ☐9 Wiskott-Aldrich Syndrome
- ☐10 Other (please specify) ______________________

6a. Did the patient experience repeated, serious, or unusual infections prior to initial diagnosis as immune deficient?

- ☐1 Yes
- ☐2 No → **SKIP TO Q7**

6b. At what age (in years) did these repeated, serious, or unusual infections begin?

______ Age of onset
☐00 Less than one year of age

7. How many times had he/she been hospitalized **before diagnosis** as immune deficient?

______ Number of times
☐00 None

8. By the time of initial diagnosis as immune deficient, had he/she suffered any **permanent impairment or loss of** ...?
SELECT ALL THAT APPLY

- ☐1 Digestive function
- ☐2 Hearing
- ☐3 Kidney function
- ☐4 Liver function
- ☐5 Lung function
- ☐6 Mobility
- ☐7 Neurological function
- ☐8 Vision
- ☐9 Other (specify) ______________________
- ☐10 No permanent loss

THE NEXT QUESTIONS ARE ABOUT THE PATIENT'S HEALTH IN THE 12 MONTHS PRIOR TO BEING DIAGNOSED AS PRIMARY IMMUNODEFICIENT

9. Would you describe his/her health in the **12 months prior to diagnosis** as...

- ☐1 Excellent
- ☐2 Very good
- ☐3 Good
- ☐4 Fair
- ☐5 Poor
- ☐6 Very poor

10. During the **12 months prior to diagnosis**, did he/she have …

SELECT ALL THAT APPLY

- ☐1 Asthma
- ☐2 Arthritis
- ☐3 Autoimmune condition
- ☐4 Cancer/leukemia
- ☐5 COPD
- ☐6 Digestive disease
- ☐7 Hepatitis
- ☐8 Malabsorption/Diarrhea
- ☐9 Lymphopenia (low white count)
- ☐10 Neurological disease
- ☐11 Neutropenia
- ☐12 Other chronic condition (please specify)

____

11. Did the patient experience any of the following infections during **the 12 months prior to diagnosis?**

Infection type	Yes	How many times
a. Abscess	☐1 →	________
b. Bronchitis	☐1 →	________
c. Candida (thrush)	☐1 →	________
d. Diarrhea (repeated)	☐1 →	________
e. Ear infection (repeated)	☐1 →	________
f. Eye infection	☐1 →	________
g. Pneumonia	☐1 →	________
h. Sepsis (blood poisoning)	☐1 →	________
i. Sinusitis	☐1 →	________
j. Skin infection	☐1 →	________
k. Other infections	☐1 →	________
l. None of these	☐1	

12. During the **12 months prior to diagnosis**, how much was he/she limited in work, play or normal physical activity as a result of his/her health.

- ☐1 No limitation
- ☐2 Slight limitation
- ☐3 Moderate limitation
- ☐4 Severe limitation

13a. Was he/she hospitalized overnight or longer for any reason during the **12 months prior to diagnosis**?

- ☐1 Yes
- ☐2 No ——→ **SKIP TO Q14**

13b. How many TIMES was he/she hospitalized in the **12 months prior to diagnosis**?

________ Times hospitalized

13c. How many NIGHTS was he/she hospitalized in the **12 months prior to diagnosis**?

________ Nights hospitalized

13d. How many NIGHTS, if any, was he/she in an INTENSIVE CARE UNIT in the **12 months prior to diagnosis?**

________ Nights in ICU

☐00 None

14. How many operations, if any, did he/she have in the **12 months prior to diagnosis**?

________ Number inpatient

________ Number outpatient

☐00 None

15. In the **12 months prior to diagnosis**, approximately how many days did he/she use..

________ Antibiotics

________ Other prescription drugs (not Ig)

________ Respiratory therapy

________ Oxygen

________ Physical therapist

________ Visiting nurse (not for Ig)

16. Not counting hospitalizations, about how many DOCTOR VISITS did the patient make during the **12 months prior to diagnosis**?

________ Primary care visits

________ Specialist visits

☐00 No doctor visits

17. Not counting hospitalizations, how many days was he/she too sick to work, go to school or perform usual activities in the **12 months prior to diagnosis**? **(parent/caregiver how many days missed due to patient illness**)

_______ Days missed work
_______ Days missed school
☐000 None
☐999 Infant/Not applicable

THIS SECTION IS ABOUT THE PATIENT'S TREATMENT EXPERIENCES

18a. Which type of physician is responsible for the treatment and management of the patient's PI?

☐1 Immunologist
☐2 Hematologist
☐3 Ear, nose & throat (ENT)
☐4 Allergist
☐5 Pulmonologist
☐6 Other (please specify)
____

18b. How many times over the past 12 months has the patient seen this physician?

_______ Times

19a. Has the patient EVER been treated with intravenous immunoglobulin (IVIG), subcutaneous immunoglobulin (SCIG) therapy or intramuscular (IM) immunoglobulin therapy on a regular basis?
SELECT ALL THAT APPLY

☐1 IVIG → **SKIP TO Q20**
☐2 SCIG → **SKIP TO Q20**
☐3 IM → **SKIP TO Q20**
☐4 No, neither → **CONTINUE**

19b. Is there any reason why the patient has never been treated with immunoglobulin replacement therapy?

☐1 Never prescribed by the doctor
☐2 Lack of insurance or inadequate insurance
☐3 Cost
☐4 Concerns about safety/side-effects
☐5 Fear of treatment
☐6 Other

IF NEVER USED SKIP TO Q71 ON PAGE 10

20. How many years, in total, has he/she been treated for immunodeficiency with IVIG, SCIG or IM (Ig therapy) on a regular basis?

_______ Years on immunoglobulin

21a. Is he/she currently being treated with SCIG, IVIG or IM for his/her immunodeficiency disease?

☐1 Yes, SCIG → **SKIP TO Q22**
☐2 Yes, IVIG → **SKIP TO Q29a**
☐3 Yes, IM → **SKIP TO Q29a**
☐4 No → **CONTINUE**

21b. Why is the patient no longer being treated with immunoglobulin?
SELECT ALL THAT APPLY

☐1 Immunoglobulin no longer prescribed by the doctor as medically necessary
☐2 Lack of insurance coverage/inadequate insurance
☐3 Too expensive (despite good insurance)
☐4 IVIG not available or hard to get
☐5 Safety/side effects
☐6 Other (PLEASE SPECIFY)
____

IF NOT USING IVIG, SCIG OR IM SKIP TO Q71 ON PAGE 10

22. What year did you start SCIG?

_______ (please enter year)

23a. How difficult was it **learning** to administer SCIG?

☐1 Very difficult
☐2 Somewhat difficult
☐3 Not too difficult
☐4 Easy

23b. How difficult is it to **administer** SCIG?

☐1 Very difficult
☐2 Somewhat difficult
☐3 Not too difficult
☐4 Easy

24. When was the last time a health professional, such as a nurse or doctor, observed and evaluated the patient's SCIG infusion technique?

- ☐1 Less than six months
- ☐2 6-12 months
- ☐3 1 year or longer

25. What is the brand name of the SCIG pump the patient uses?

____

26. Prior to SCIG therapy did the patient receive IVIG therapy?

- ☐1 Yes → **CONTINUE**
- ☐2 No → **SKIP TO Q30**

27. Why did the patient switch from IVIG to SCIG? **SELECT ALL THAT APPLY**

- ☐1 Insurance reasons
- ☐2 Physician recommended it
- ☐3 Problems finding a vein for IVIG
- ☐4 Patient is an infant/child
- ☐5 Problems getting to IVIG infusion site
- ☐6 Have limited mobility
- ☐7 It is more convenient
- ☐8 Reactions to IVIG
- ☐9 Other (specify below)

____

28. Compared to IVIG therapy how well do you feel SCIG therapy controls the patient's PI?

- ☐1 Much better than IVIG
- ☐2 Better than IVIG
- ☐3 About the same as IVIG
- ☐4 Worse than IVIG
- ☐5 Much worse than IVIG

CURRENT SCIG USER SKIP TO Q30

29a. Has the patient ever been on SCIG therapy?

- ☐1 Yes
- ☐2 No → **SKIP TO Q30**

29b. Why is the patient no longer on SCIG therapy?

- ☐1 Had bad side-effects
- ☐2 Had a problem using needles
- ☐3 Had a problem with the pump
- ☐4 Too long to infuse
- ☐5 Cost too much
- ☐6 Did not control PI as well as IVIG
- ☐7 Dosing was too frequent
- ☐8 Self-administration was difficult
- ☐9 Insurance would not pay for SCIG
- ☐10 Reminds patient of their disease
- ☐11 Other ____________________

30. On average, how often does he/she get their IVIG, SCIG or IM therapy?

- ☐1 Daily
- ☐2 Three times per week
- ☐3 Two times per week
- ☐4 Every week
- ☐5 Every two weeks
- ☐6 Every three weeks
- ☐7 Every four weeks
- ☐8 Every five weeks
- ☐9 Every six weeks or more

31. About how many grams of Ig per infusion does he/she normally receive?

________ Grams

________ mL (if SCIG)

☐888 Not sure

32. Who usually administers the therapy?

- ☐1 Doctor
- ☐2 Nurse
- ☐3 Patient (self-infused)
- ☐4 Other family member
- ☐5 Other

33. Where does the patient usually receive his/her Ig therapy?

- ☐1 At home, self-infused
- ☐2 At home, nurse infused
- ☐3 Doctor's private office
- ☐4 Hospital outpatient
- ☐5 Hospital clinic
- ☐6 Infusion suite
- ☐7 Other (specify)

____

34. How long does the therapy usually take?

_______ Hours (a)
_______ Minutes (b)

35. About how much does the patient weigh?

_______ Weigh in pounds

36. Who determines the rate of infusion?

☐1 Patient/parent
☐2 Doctor
☐3 Nurse
☐4 Other (please specify)

____

37a. Is he/she given medication before Ig therapy, like an antihistamine, cortico-steroid or anti-inflammatory like Tylenol or Motrin, to make it go easier or faster?
SELECT ALL THAT APPLY

	By mouth	IV
a. Antihistamine, usually	☐1	☐2
b. Antihistamine, sometimes	☐1	☐2
c. Steroid, usually	☐1	☐2
d. Steroid, sometimes	☐1	☐2
e. Never any of these	☐1	

38a. Does the patient (parent/caretaker) need to take off from school or work to get their Ig therapy?

☐1 Yes
☐2 No ⟶ **SKIP TO Q39**

38b. How many days **in the past 12 months** has the patient (parent/caretaker) needed to take off work or miss school to get their Ig therapy?

_______ Days missed school

_______ Days missed work

39. **During the past 12 months**, has he/she experienced any of the following **during or after** Ig therapy?
SELECT ALL THAT APPLY

	During	After
a. Abdominal pain	☐1	☐2
b. Anxiety	☐1	☐2
c. Blood in tubing (SCIG)	☐1	☐2
d. Chills	☐1	☐2
e. Diarrhea	☐1	☐2
f. Dizziness	☐1	☐2
g. Fatigue	☐1	☐2
h. Fever	☐1	☐2
i. Headache	☐1	☐2
j. Hepatitis	☐1	☐2
k. Hives	☐1	☐2
l. Increase in blood pressure	☐1	☐2
m. Migraine headache	☐1	☐2
n. Muscle spasms	☐1	☐2
o. Nausea	☐1	☐2
p. Drop in blood pressure	☐1	☐2
q. Swelling at infusion site	☐1	☐2
r. Redness at infusion site	☐1	☐2
s. Muscle aches	☐1	☐2
t. Kidney problems	☐1	☐2
u. Vomiting	☐1	☐2
v. Weakness	☐1	☐2
w. Wheezing	☐1	☐2

40a. Has the patient ever had any of the following **serious side-effects or reactions** from their Ig therapy?
PLEASE SELECT ALL THAT APPLY

☐1 Aseptic meningitis
☐2 Blood clots
☐3 Blurred vision
☐4 Hemolytic anemia
☐5 Pulmonary embolism
☐6 Seizure
☐7 Stroke
☐8 NONE OF THESE ⟶ **SKIP TO Q41a**

40b. When was the most recent time that he/she had a **serious side effect or reaction** from their Ig?

- ☐1 0 to 6 months
- ☐2 7 to 12 months
- ☐3 1 to 2 years ago
- ☐4 3 to 4 years ago
- ☐5 5 years or more
- ☐6 Never ⟶ **SKIP TO Q41a**

40c. Did the patient's side effect or reaction cause him/her to ...
SELECT ALL THAT APPLY

- ☐1 Slow down infusion rate
- ☐2 Switch products
- ☐3 Prefer a specific Ig product
- ☐4 Only receive Ig in doctor's office
- ☐5 Report the event to the FDA
- ☐6 Other (specify) ____________________
- ☐7 None of these

40d. Was the doctor told about the patient's serious side-effect or reaction?

- ☐1 Yes ⟶ **CONTINUE**
- ☐2 No ⟶ **SKIP TO Q40F**

40e. What did the doctor do?

- ☐1 Switched products
- ☐2 Reduced amounts
- ☐3 Slowed infusion rate
- ☐4 Gave medicine
- ☐5 Said it was normal
- ☐6 Changed from IVIG to SCIG
- ☐7 Changed from SCIG to IVIG
- ☐8 Nothing

40f. Has the patient ever had a serious side effect or reaction from Ig therapy when...
SELECT ALL THAT APPLY

- ☐1 Trying a new product for the first time
- ☐2 Switched to a different product used before
- ☐3 Using a product with no previous problems
- ☐4 None of these

41a. Has the patient (parent/caretaker of a patient) ever missed school or work **due to a reaction from** their Ig therapy?

- ☐1 Yes
- ☐2 No ⟶ **SKIP TO Q42**

41b. How many days in the past 12 months has the patient (parent/caretaker of a patient) missed school or work due to reactions from Ig therapy?

________ Days missed school

________ Days missed work

42. How well does immunoglobulin control the patient's immunodeficiency?

- ☐1 Completely controlled
- ☐2 Well controlled
- ☐3 Adequately controlled
- ☐4 Less than adequately controlled
- ☐5 Poorly controlled

43a. Does the patient experience periods of fatigue or low energy between Ig therapy treatments (wear off)?

- ☐1 Always ⟶ **CONTINUE**
- ☐2 Occasionally ⟶ **CONTINUE**
- ☐3 Never ⟶ **SKIP TO Q44**

43b. How long after infusion does he/she feel this wear off?

________ Days after infusion

43c. Does the "wear off" result in any of the following? **SELECT ALL THAT APPLY**

- ☐1 Infection
- ☐2 Need for antibiotics
- ☐3 Need for other medication
- ☐4 Missed school or work
- ☐5 Forgoing usual activity
- ☐6 Forgoing of a pleasurable activity
- ☐7 Decreased performance in usual activities

44. Does he/she tolerate any immunoglobulin products better than others, or are they all about the same?

- ☐1 All about the same
- ☐2 Some better than others
- ☐3 Have only been on one product

45. As a result of concerns about product tolerability has he/she ever …
SELECT ALL THAT APPLY

- ☐1 Refused a particular product
- ☐2 Switched off a product
- ☐3 Switched to another product
- ☐4 Delayed a scheduled infusion
- ☐5 None of these

46. As a result of product effectiveness has he/she ever…
SELECT ALL THAT APPLY

- ☐1 Refused a particular product
- ☐2 Switched off a product
- ☐3 Switched to another product
- ☐4 Delayed a scheduled infusion
- ☐5 None of these

47a. Which of the following Ig products has the patient ever used?

47b. Which of the following Ig products do they **currently** use?

Product	47b ↓	47a ↓
a. Bivigam (Biotest)	☐1	☐2
b. Carimune (CSL Behring)	☐1	☐2
c. Flebogamma (Grifols)	☐1	☐2
d. Gammagard Liquid (Baxter)	☐1	☐2
e. Gammagard S/D (Baxter)	☐1	☐2
f. Gammaked (Kedrion)	☐1	☐2
g. Gamunex-C (Grifols)	☐1	☐2
h. Gammaplex (Bio Products)	☐1	☐2
i. Hizentra (CSL Behring)	☐1	☐2
j. Octagam (Octapharma)	☐1	☐2
k. Privigen (CSL Behring)	☐1	☐2
l. Other	☐1	☐2

48. How often does he/she get the Ig product they prefer most?

- ☐1 Always
- ☐2 Most of the time
- ☐3 Some of the time
- ☐4 Only occasionally
- ☐5 Never
- ☐6 Only tried one product

49. How satisfied is the patient with the Ig product **currently** being used?

- ☐1 Very satisfied
- ☐2 Somewhat satisfied
- ☐3 Neither
- ☐4 Somewhat dissatisfied
- ☐5 Very dissatisfied

50. Who is primarily responsible for the selection of the Ig product that the patient uses?

- ☐1 Patient
- ☐2 Doctor
- ☐3 Medical plan/facility
- ☐4 Insurance provider
- ☐5 Other (please specify)

____

51a. Thinking about the patient's experience with their **current** Ig therapy, please tell us how much the patient is bothered, if at all, by each of the following.

	Not bothered at all ↓	Bothered a little bit ↓	Moderately bothered ↓	Bothered quite a bit ↓	Extremely Bothered ↓
a. Convenience of treatment	☐1	☐2	☐3	☐4	☐5
b. Severe side-effects	☐1	☐2	☐3	☐4	☐5
c. Minor side-effects	☐1	☐2	☐3	☐4	☐5
d. Local site reactions	☐1	☐2	☐3	☐4	☐5
e. Number of needle sticks	☐1	☐2	☐3	☐4	☐5
f. Time to infuse	☐1	☐2	☐3	☐4	☐5
g. Number of infusions each month	☐1	☐2	☐3	☐4	☐5
h. Cost of infusions	☐1	☐2	☐3	☐4	☐5
i. Interrupts life	☐1	☐2	☐3	☐4	☐5
j. Operating pump or infusion device	☐1	☐2	☐3	☐4	☐5
k. Other____________	☐1	☐2	☐3	☐4	☐5

51b. Overall, how bothered is the patient when they get Ig therapy?

☐1 Not bothered at all
☐2 Bothered a little bit
☐3 Moderately bothered
☐4 Bothered quite a bit
☐5 Extremely bothered

THE NEXT QUESTIONS ARE ABOUT THE PATIENT'S HEALTH IN THE PAST 12 MONTHS.

52. In general would you say the patient's health is:

☐1 Excellent
☐2 Very good
☐3 Good
☐4 Fair
☐5 Poor
☐6 Very poor

53. During the **past 12 months**, has he/she had:

☐1 Asthma
☐2 Arthritis
☐3 Autoimmune condition
☐4 Cancer/leukemia
☐5 COPD
☐6 Digestive disease
☐7 Hepatitis
☐8 Malabsorption/diarrhea
☐9 Lymphopenia (low white count)
☐10 Neurological disease
☐11 Neutropenia
☐12 Other chronic condition

____

54. Did he/she experience the following infections during the **past 12 months**?

Infection type	Yes	How many times
a. Abscess	☐1 →	______
b. Bronchitis	☐1 →	______
c. Candida (thrush)	☐1 →	______
d. Diarrhea (repeated)	☐1 →	______
e. Ear infection (repeated)	☐1 →	______
f. Eye infection	☐1 →	______
g. Pneumonia	☐1 →	______
h. Sepsis (blood poisoning)	☐1 →	______
i. Sinusitis	☐1 →	______
j. Skin infection	☐1 →	______
k. Other infections	☐1 →	______
l. None of these	☐1	

55. During the **past 12 months**, how much has he/she been limited in work, play or normal physical activity as a result of his/her health?

☐1 No limitation
☐2 Slight limitation
☐3 Moderate limitation
☐4 Severe limitation

56a. Has he/she been hospitalized overnight or longer for any reason during the **past 12 months**?

☐1 Yes
☐2 No → **SKIP TO Q57**

56b. How many TIMES was he/she hospitalized in the **past 12 months**?

________ Times hospitalized

56c. How many NIGHTS was he/she hospitalized in the **past 12 months**?

________ Nights hospitalized

56d. How many NIGHTS, if any, was he/she in an INTENSIVE CARE UNIT in the **past 12 months**?

________ Nights in ICU

☐00 None

57. How many operations, if any, did he/she have in the past **12 months**?

________ Number inpatient
________ Number outpatient

☐00 None

58. Approximately how many days in the **past 12 months** did he/she use:

________ Antibiotics
________ Other prescription drugs (not Ig)
________ Respiratory therapy
________ Oxygen
________ Physical therapist
________ Visiting nurse (not for Ig)

59. Not counting hospitalizations, about how many doctor visits did the patient make during the **past 12 months**?

________ Primary care visits
________ Specialist visits

☐00 No doctor visits

60. Not counting hospitalizations, how many days was he/she too sick to work, go to school or perform usual activities in the past **12 months**? **(parent/caregiver how many days missed due to patient illness)**

_________ Days missed work
_________ Days missed school

☐000 None
☐999 Infant/Not applicable

61. In the **past 12 months**, how long has the patient taken antibiotics **to prevent infections (prophylactically)**?

☐1 Less than 1 month
☐2 1 to 6 months
☐3 Longer than 6 months
☐4 Did not take any antibiotics → **SKIP TO Q62**

(please list antibiotics below)

____
____
____

62. In the **past 12 months**, how long has the patient taken antibiotics **for an active infection**?

☐1 Less than 1 month
☐2 1 to 6 months
☐3 Longer than 6 months
☐4 Did not take any antibiotics → **SKIP TO Q63**

(please list antibiotics below)

____
____
____

63. What kind of health insurance does the **patient** currently have?
SELECT ALL THAT APPLY

☐1 Employer sponsored group plan
☐2 COBRA
☐3 Individual policy
☐4 Medicare A & B
☐5 Medicare Supplemental Plan
☐6 Medicare Advantage Plan
☐7 Medicare due to disability
☐8 Medicaid
☐9 SCHIP or other government policy
☐10 State Exchange/Marketplace
☐11 Federal Exchange/Marketplace
☐12 TRICARE
☐13 Veterans Policy
☐14 Other Insurance (specify)

____

☐15 No health insurance coverage

64. In the past three years, **due to health insurance**, has the patient ever had a problem in getting his/her regular infusion?

☐1 Yes
☐2 No ⟶ **SKIP TO Q66**

65. When was the most recent time the patient had a problem getting his/her regular infusion **due to health insurance?**

☐1 Past month
☐2 Past six months
☐3 Past year
☐4 Two to three years ago

66. In **the past 12 months**, which of the following problems, if any, has the patient experienced **due to health insurance**?
SELECT ALL THAT APPLY

☐1 Site of care for infusion changed
☐2 Increased interval between infusion
☐3 Reduced dosage of infusion
☐4 Delayed infusions
☐5 Cancelled infusions
☐6 Switched to less tolerated product
☐7 Switched to less preferred product
☐8 Switched from IVIG to SCIG
☐9 Switched from SCIG to IVIG
☐10 No product available
☐11 Reimbursement problems
☐12 Treating physicians now out of network
☐13 Any other problem (specify)

____

☐14 None

67. How many times in the **past 12 months** has the patient experienced a problem getting his/her regular infusion?

________ times in past 12 months

☐888 None

68. As a result of health insurance policies, has the patient had any problems seeing healthcare specialists?

☐1 Yes
☐2 No

69. Since December 2012, what changes, if any, have you experienced with your **overall** health insurance costs?

☐1 I pay MORE for my health insurance
☐2 I pay LESS for my health insurance
☐3 I pay the SAME for my health insurance

70. Specifically thinking about your **personal costs** for Ig replacement therapy, **since December 2012** what changes, if any, have you experienced with paying for Ig replacement therapy?

☐1 It costs me MORE for Ig therapy
☐2 It costs me LESS for Ig therapy
☐3 It costs me the SAME for Ig therapy

71. As you may know, a health reform bill known as the Affordable Care Act (ACA) was signed into law in 2010. Given what you know about the ACA, which of the following statements most closely matches your view.

"Within the next 12 months I will be…"

☐1 **Voluntarily** enrolled in the Health Insurance Marketplace
☐2 **Forced** to enroll in the Health Insurance Marketplace
☐3 Insured through an employer
☐4 Insured through Medicare
☐5 Insured through Medicaid
☐6 Not sure how I will get my health insurance

THE LAST FEW QUESTIONS ARE TO HELP IDF LEARN MORE ABOUT WHO IS AFFECTED BY PRIMARY IMMUNODEFICIENCY DISEASES.

72. Which of the following categories would best describe the race or ethnicity of the patient?

☐1 American Indian/Alaskan native
☐2 Asian/Pacific Islander
☐3 Black/African-American
☐4 Hispanic or Latino
☐5 White, non-Hispanic
☐6 Two or more races
☐7 Other (Specify)

____

PLEASE TURN PAGE

73. What is the current employment status of the **patient** (head of household if patient is a child)?

- ☐1 Employed full time
- ☐2 Employed part time
- ☐3 Unemployed, looking for work
- ☐4 Student
- ☐5 Homemaker
- ☐6 Disabled/too ill to work
- ☐7 Other

74. What is the last grade or year of school completed by the **patient** (head of household if patient is a child)?

- ☐1 8th grade or less
- ☐2 Some high school
- ☐3 High school grad/GED
- ☐4 1-3 years of college
- ☐5 4 year college grad
- ☐6 Graduate degree

75. What was the patient's (or household's) total income last year?

- ☐1 0 to $24,999
- ☐2 $25,000 to $49,999
- ☐3 $50,000 to $74,999
- ☐4 $75,000 to $99,000
- ☐5 $100,000 or more

PLEASE RETURN IN THE ENCLOSED ENVELOPE TO THE IMMUNE DEFICIENCY FOUNDATION

IF YOU HAVE ANY QUESTIONS ABOUT THIS SURVEY PLEASE CALL THE IMMUNE DEFICIENCY FOUNDATION AND ASK FOR THE DIRECTOR OF SURVEY RESEARCH 1.800.296.4433

Adult patients - Please complete the survey *SF-12 Your Health and Well Being.*

Adult patients with PI with a child with PI - Please complete the survey *SF-12 Your Health and Well Being.*

Parents of a child with PI - Please complete the survey *SF-10 Health Survey for Children* **only** if your child is between the ages of 5 and 18.
