# Supplementary figures and images for: Health-Related Quality of Life in Adult Patients with Common Variable Immunodeficiency Disorders and Impact of Treatment
Source: J Clin Immunol. 2017 May 23;37(5):461–75. doi: 10.1007/s10875-017-0404-8 (PMC5489588; doi:10.1007/s10875-017-0404-8)

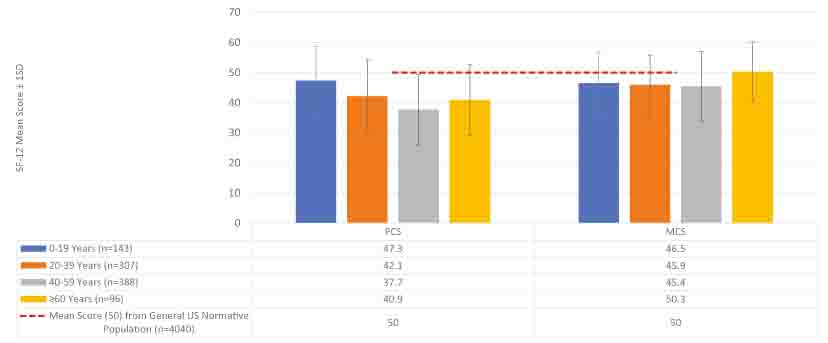

Supplement: Supplementary file 2 — (JPEG 26 kb). [file 10875_2017_404_Fig7_ESM.jpg]

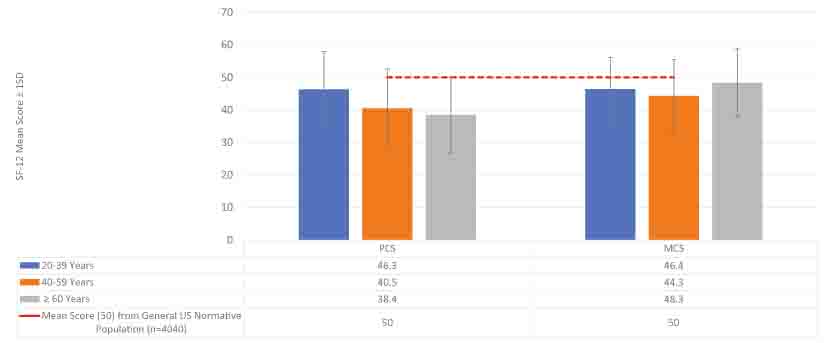

Supplement: Supplementary file 4 — (JPEG 25 kb). [file 10875_2017_404_Fig8_ESM.jpg]

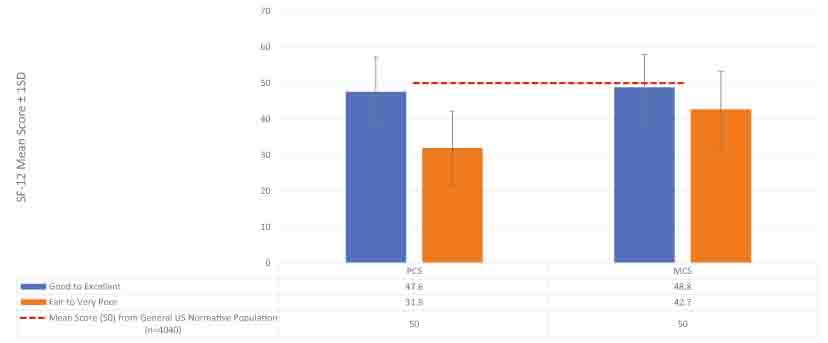

Supplement: Supplementary file 6 — (JPEG 24 kb). [file 10875_2017_404_Fig9_ESM.jpg]

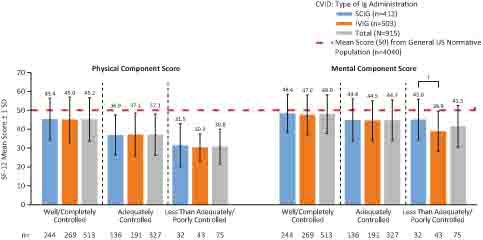

Supplement: Supplementary file 8 — (JPEG 41 kb). [file 10875_2017_404_Fig10_ESM.jpg]

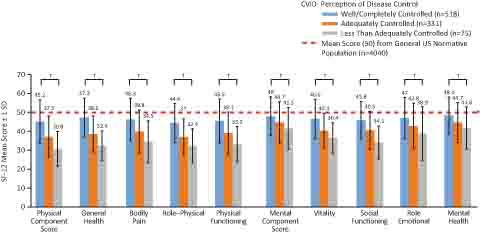

Supplement: Supplementary file 10 — (JPEG 41 kb). [file 10875_2017_404_Fig11_ESM.jpg]

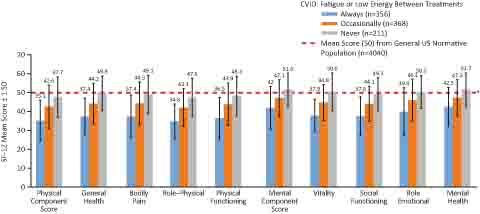

Supplement: Supplementary file 12 — (JPEG 40 kb). [file 10875_2017_404_Fig12_ESM.jpg]

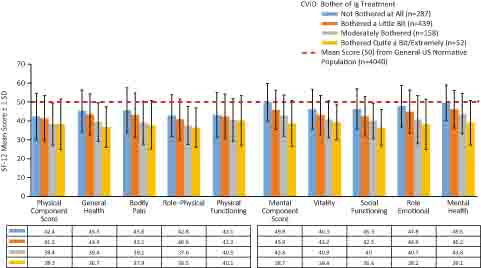

Supplement: Supplementary file 14 — (JPEG 47 kb). [file 10875_2017_404_Fig13_ESM.jpg]

## Unadjusted model

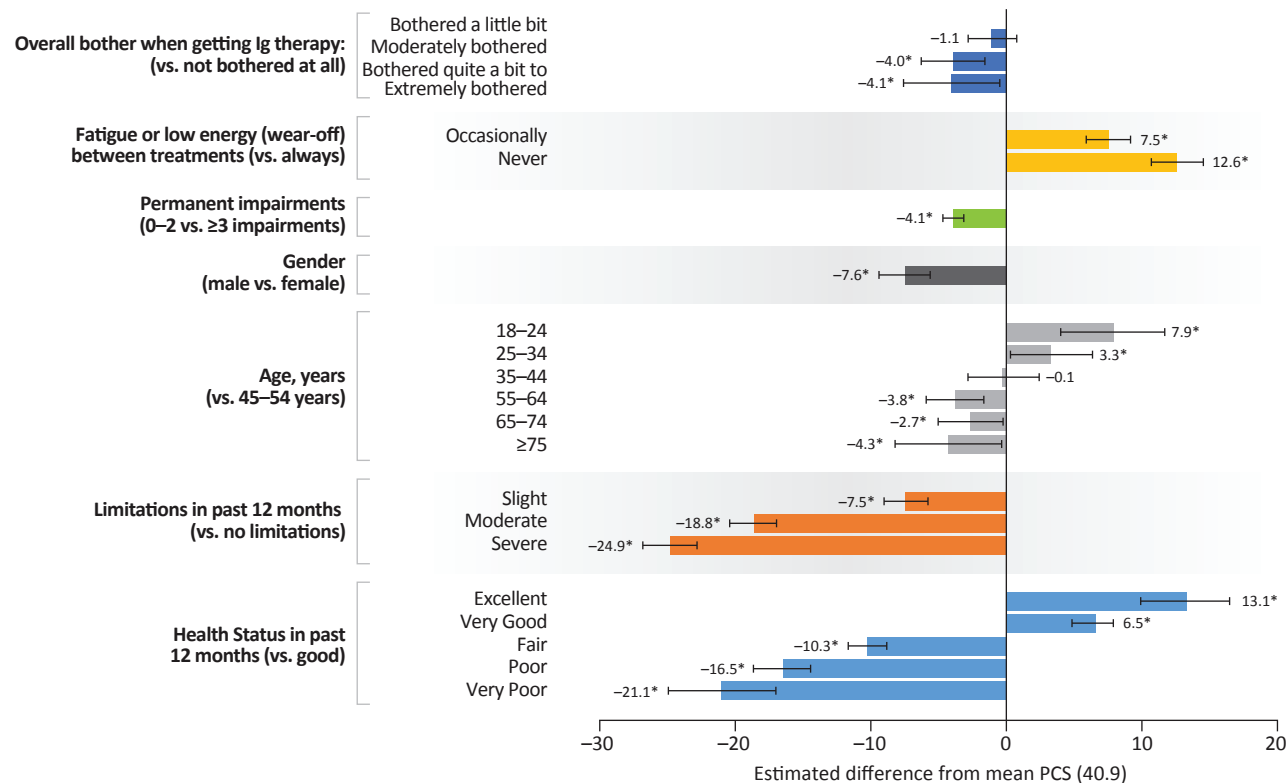

## Adjusted model

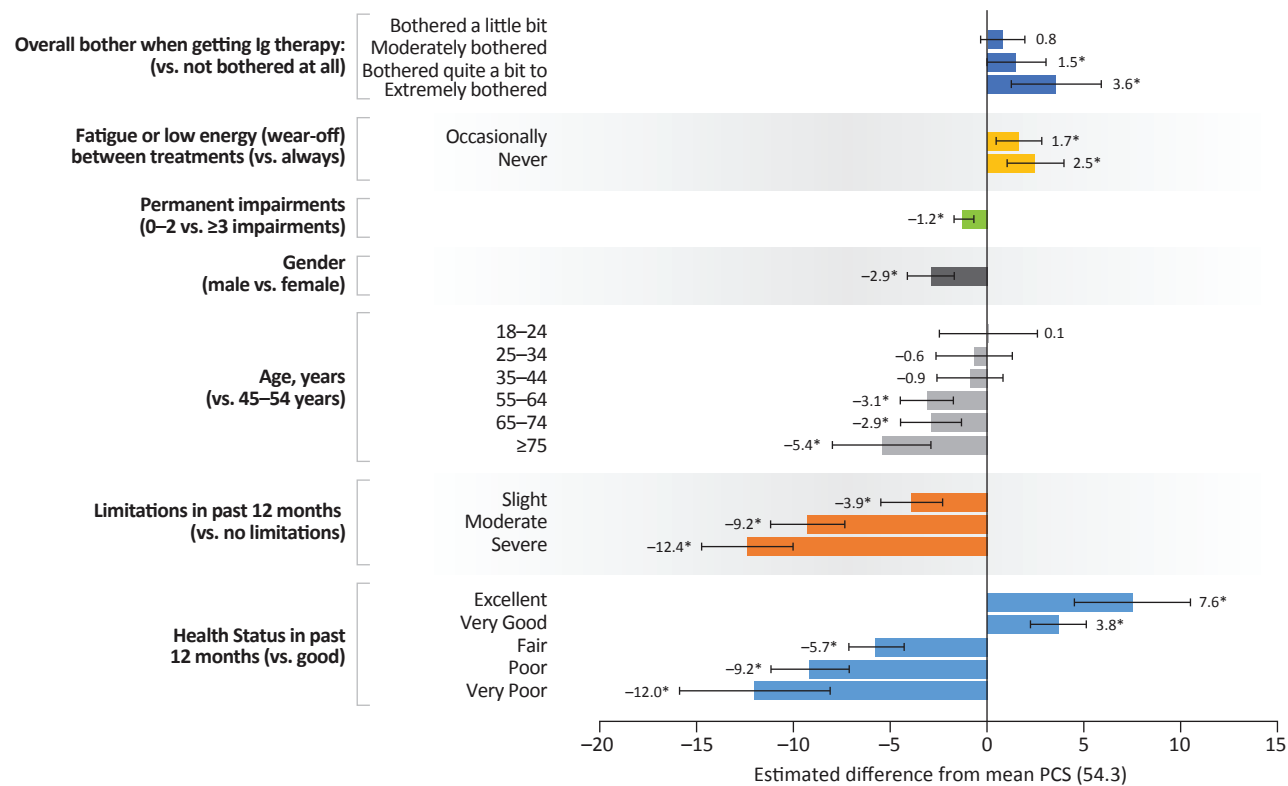

Supplement: Supplementary file 16 — (PDF 108 kb). [file 10875_2017_404_MOESM9_ESM.pdf]

## Unadjusted model

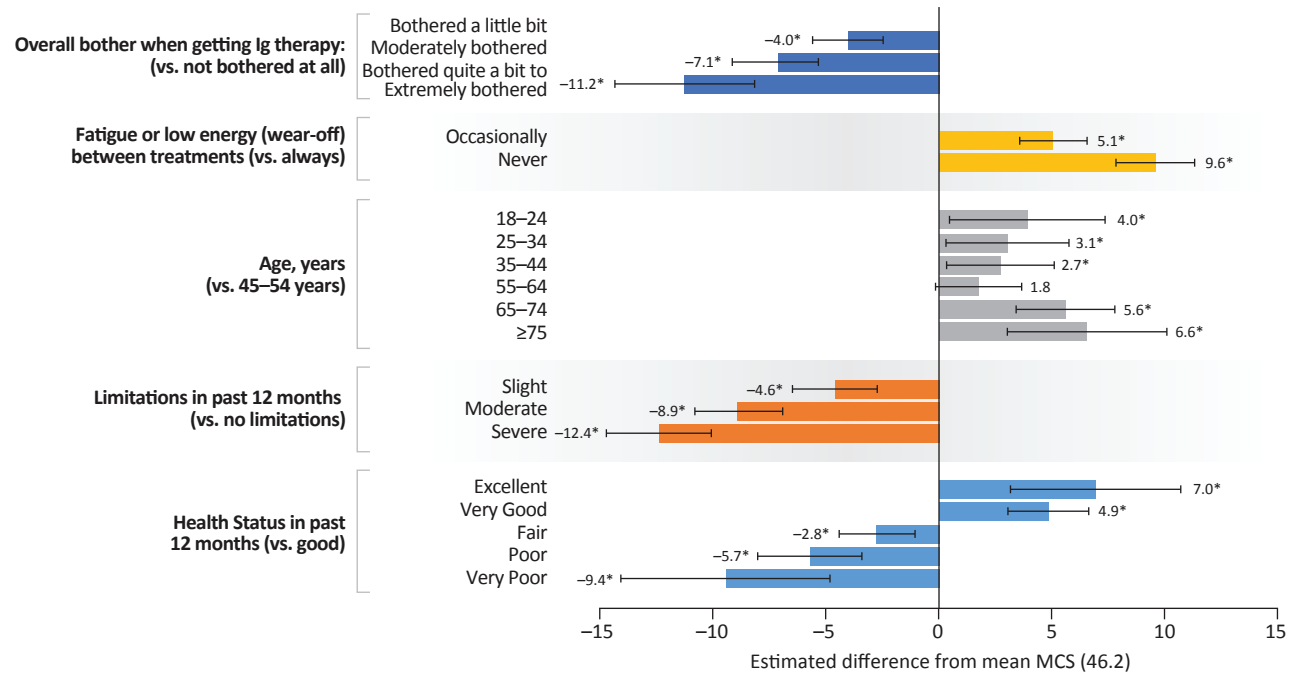

## Adjusted model

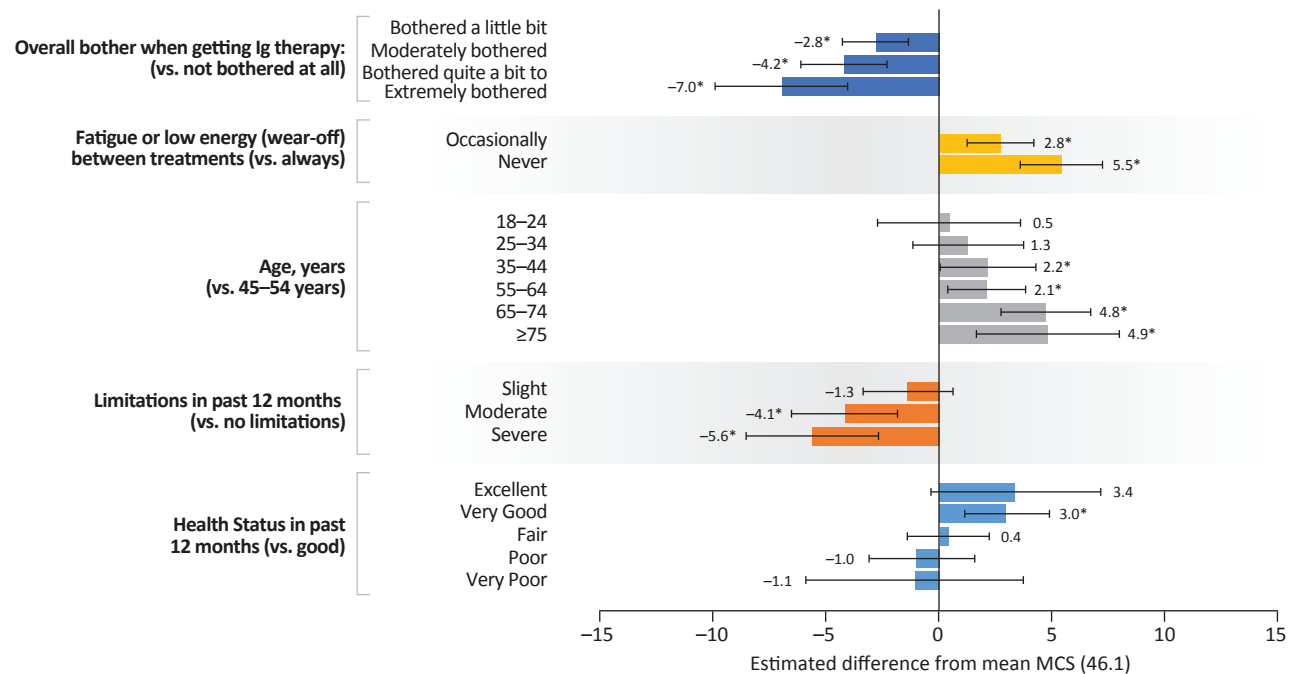

Supplement: Supplementary file 17 — (PDF 109 kb). [file 10875_2017_404_MOESM10_ESM.pdf]
