# Supplementary material for: Health-Related Quality of Life in Adult Patients with Common Variable Immunodeficiency Disorders and Impact of Treatment
Source: J Clin Immunol. 2017 May 23;37(5):461–75. doi: 10.1007/s10875-017-0404-8 (PMC5489588; doi:10.1007/s10875-017-0404-8)
Supplement: Supplementary file 18 — (DOCX 200 kb). [file 10875_2017_404_MOESM11_ESM.docx]

**Supplemental Data**

**Health Related Quality of Life in Adult Patients with Common Variable Immunodeficiency and the Impact of Treatment**

*Journal of Clinical Immunology*

Nicholas L. Rider · Carleigh Kutac · Joud Hajjar · Chris Scalchunes · Filiz O. Seeborg · Marcia Boyle · Jordan S. Orange

**Correspondence:** Dr. Nicholas L. Rider, D.O., Section of Immunology, Allergy and Rheumatology, Texas Children’s Hospital, 1102 Bates St, Suite 330, Houston, TX, 77030, USA
E-mail: nlrider@bcm.edu

**Fig. S1** Immune Deficiency Foundation 2013 treatment survey


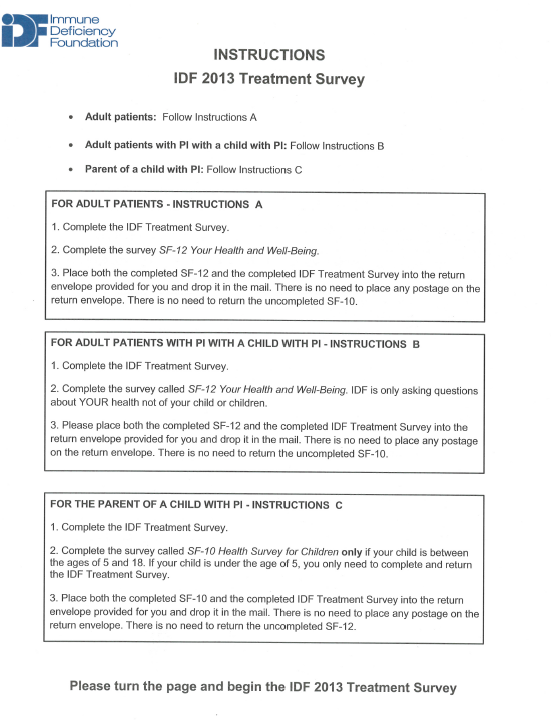


**Fig. S2** SF-12 mean Physical^a^ and Mental^b^ Component and Domain Scores for patients with CVID, by age diagnosed

Please see attached figure.

^*^ Significant difference compared with the general US population normative sample (*p*<0.05; lower than US norm)

^a^ Domains of the Physical Component Score: general health, bodily pain, role–physical, and physical functioning

^b^ Domains of the Mental Component Score: vitality, social functioning, role–emotional, and mental health

*CVID* common variable immunodeficiency, *SD* standard deviation, *SF-12* 12-item Short Form Health Survey

**Fig. S3** SF-12 mean Physical^a^ and Mental^b^ Component and Domain Scores for patients with CVID, by consolidated age group

Please see attached figure.

^a^ Domains of the Physical Component Score: general health, bodily pain, role–physical, and physical functioning

^b^ Domains of the Mental Component Score: vitality, social functioning, role–emotional, and mental health

Significant differences in SF-12 scores between:

^*^ Age 20–39 vs 40–59 years (*p*<0.05) for all categories

^†^ Age 20–39 vs ≥60 years (*p*<0.05) for PCS, MCS, and all domain groups, with the exception of role–emotional and mental health

^‡^ Age 40–59 vs ≥60 years (*p*<0.05) for PCS and physical functioning

*CVID* common variable immunodeficiency, *MCS* Mental Component Score, *PCS* Physical Component Score, *SD* standard deviation, *SF-12* 12-item Short Form Health Survey, *US norm* general US population normative sample

**Fig. S4** SF-12 mean Physical^a^ and Mental^b^ Component Scores for patients with CVID, by health in the past 12 months^c^

Please see attached figure.

**^*^** Significant differences in PCS and MCS between the general US population normative sample and patients considering their health <12 months prior to diagnosis (different/lower than US norm, p < 0.001).

*CVID* common variable immunodeficiency, *SD* standard deviation, *SF-12* 12-item Short Form Health Survey

**Fig. S5** SF-12 mean Physical and Mental Component Scores for patients with CVID: perception of disease control, by route of Ig administration^a^

Please see attached figure.

^*^ Significant difference between all categories compared with a US normative population (*p*<0.05; lower than US norm)

^†^ Significant difference between SCIG and IVIG (*p*<0.05)

^a^ Between-group comparison for PCS and MCS: Significant difference between “well controlled” vs “adequately controlled” (*p*<0.05) and “less than adequately/poorly controlled” (*p*<0.05) PIDD; significant difference between “adequately controlled” vs “less than adequately/poorly controlled” (*p*<0.05) PIDD

*CVID* common variable immunodeficiency, *Ig* immunoglobulin, *IVIG* intravenous administration of Ig, *SCIG* subcutaneous administration of Ig, *SD* standard deviation, *SF-12* 12-item Short Form Health Survey

**Fig. S6** SF-12 Mean Physical^a^ and Mental^b^ Component and Domain Scores for patients with CVID: perception of disease control

Please see attached figure.

^*^ Significant difference in mean SF-12 scores between all categories compared with the general US population normative sample (*p*<0.05; lower than US norm)

^†^ Significant difference in SF-12 (all categories) between patients with perceived “well/completely controlled” vs “adequately controlled” or “less than adequately controlled” disease (*p*<0.05) and “adequately controlled” vs “less than adequately controlled” disease (*p*<0.05)

^a^ Domains of the Physical Component Score: general health, bodily pain, role–physical, and physical functioning

^b^ Domains of the Mental Component Score: vitality, social functioning, role–emotional, and mental health

*CVID* common variable immunodeficiency, *SD* standard deviation, *SF-12* 12-item Short Form Health Survey, *US norm* general US population normative sample **Fig. S7** SF-12 mean Physical^a^ and Mental^b^ Component and Domain Scores for patients with CVID (“Does the patient experience periods of fatigue or low energy between Ig treatments?”)^c^

Please see attached figure.

^*^ Significant differences in SF-12 between the general US normative population and patients who “always”, “occasionally”, or “never” experienced period of fatigue or low energy between Ig treatment: “never” for PCS, physical functioning, and role–physical (*p*<0.05; lower that the US norm) and MCS (*p*<0.05; higher than the norm); “occasionally” and “always” for all categories (*p*<0.05; lower than the US norm)

^a^ Domains of the Physical Component Score: general health, bodily pain, role–physical, and physical functioning

^b^ Domains of the Mental Component Score: vitality, social functioning, role–emotional, and mental health

^c^Between-group comparisons indicated a significant difference in SF-12 scores between patients who repored “never” experiencing post-infusion fatigue and patients who reported “always” or “occasionaly” experiencing post-infusion fatigue (*p*<0.05)

*CVID* common variable immunodeficiency, *Ig* immunoglobulin, *MCS* Mental Component Score, *PCS* Physical Component Score, *SD* standard deviation, *SF-12* 12-item Short Form Health Survey, *US norm* general US population normative sample

**Fig. S8** SF-12 mean Physical^a^ and Mental^b^ Component and Domain Scores for patients with CVID, by bother of treatment^c^

Please see attached figure.

^*^ Significant differences in SF-12 between the general US normative population and patients who were “bothered a little bit” or worse (*p*<0.05) (lower than US norm all categories) and “not bothered at all” (*p*<0.05) (lower than US norm all categories with the exception of MCS and mental health)

^a^ Domains of the Physical Component Score: general health, bodily pain, role–physical, and physical functioning

^b^ Domains of the Mental Component Score: vitality, social functioning, role–emotional, and mental health

^c^ Significant difference in mean SF-12 scores between patients “not bothered at all” vs “bothered a little bit” or worse (*p*<0.05) (all categories except physical functioning and role physical) and “not bothered at all” vs “moderately bothered” or “extremely bothered” (*p*<0.05; all categories)

*CVID* common variable immunodeficiency, *Ig* immunoglobulin, *MCS* Mental Component Score, *SD* standard deviation, *SF-12* 12-item Short Form Health Survey, *US norm* general US population normative sample
