# Supplementary material for: Health-Related Quality of Life in Adult Patients with Common Variable Immunodeficiency Disorders and Impact of Treatment
Source: J Clin Immunol. 2017 May 23;37(5):461–75. doi: 10.1007/s10875-017-0404-8 (PMC5489588; doi:10.1007/s10875-017-0404-8)
Supplement: Supplementary file 19 — (DOCX 51 kb). [file 10875_2017_404_MOESM12_ESM.docx]

**Table S1** IDF CVID Cohort Patient-reported Health (*N*=945)

| Characteristic | *n* (%) |
| --- | --- |
| How well does Ig treatment control PIDD (in general)?  Well/completely controlled  Adequately controlled  Less than adequately/poorly controlled  Missing | 518 (54.8)  331 (35.0)  75 (7.9)  21 (2.2) |
| Patient experiences periods of fatigue or low energy between Ig treatment (in general)  Always  Occasionally  Never  Missing | 356 (37.7)  368 (38.9)  211 (22.3)  10 (1.1) |
| Overall, how bothered is the patient when they get their Ig treatment (in general)?^b^  All patients  Not bothered at all  Bothered a little bit  Moderately bothered  Bothered quite a bit/extremely  Missing  Patients treated with SCIG  Not bothered at all  Bothered a little bit  Moderately bothered  Bothered quite a bit/extremely  Missing  Patients treated with IVIG  Not bothered at all  Bothered a little bit  Moderately bothered  Bothered quite a bit/extremely  Missing | 287 (30.4)  439 (46.5)  158 (16.7)  52 (5.5)  9 (1.0)  105 (25.2)  214 (51.3)  79 (18.9)  19 (4.6)  7 (1.7)  181 (35.0)  224 (43.3)  79 (15.3)  33 (6.4)  2 (0.4) |
| Patient health in the past 12 months  Excellent  Very good  Good  Fair  Poor  Very poor  Missing | 31 (3.3)  202 (21.4)  303 (32.1)  263 (27.8)  99 (10.5)  20 (2.1)  27 (2.9) |
| Patient limitation^c^ in the past 12 months  None  Slight  Moderate  Severe  Missing | 164 (17.4)  335 (35.4)  291 (30.8)  135 (14.3)  20 (2.1) |
| Patient hospitalized overnight in the past 12 months  No  Yes  Missing | 723 (76.5)  206 (21.8)  16 (1.7) |
| Patient hospitalized in the intensive care unit in the past 12 months  No  Yes  Missing | 903 (95.6)  28 (3.0)  14 (1.5) |
| In-patient operation in the past 12 months^d^  No  Yes | 774 (87.7)  109 (12.3) |
| Out-patient operation in the past 12 months^d^  No  Yes | 688 (77.9)  195 (22) |

^a^ Patient reporting “permanent impairment/loss” that was not related to digestive and/or lung function

^b^ Patients treated with IMIG (*n*=2) not included in analysis

^c^ Limitations are defined as how much the patient was limited in work, play or normal physical activity as a result of his/her health (for 12 months prior to diagnosis and for the past 12 months)

^d^ Data does not reflect 62 missing responses; missing responses may reflect an actual omission of a response, or the patient did not respond because they did not have the operation

*CVID* common variable immunodeficiency, *Ig* immunoglobulin, *IMIG* intramuscular immunoglobulin, *IVIG* intravenous immunoglobulin, *PIDD* primary immunodeficiency disease, *SCIG* subcutaneous immune globulin
