# Supplementary material for: Health-Related Quality of Life in Adult Patients with Common Variable Immunodeficiency Disorders and Impact of Treatment
Source: J Clin Immunol. 2017 May 23;37(5):461–75. doi: 10.1007/s10875-017-0404-8 (PMC5489588; doi:10.1007/s10875-017-0404-8)
Supplement: Supplementary file 20 — (DOCX 54 kb). [file 10875_2017_404_MOESM13_ESM.docx]

**Table S2** SF-12 mean Physical and Mental Component Scores for various QOL parameters, compared with the general US population normative sample and within QOL categories

|  | *n* | Mental Component Score | | | Physical Component Score | | |
| --- | --- | --- | --- | --- | --- | --- | --- |
|  |  | Mean (SD) | *P*-value | | Mean (SD) | *P*-value | |
|  |  |  | vs US norm | vs within category |  | vs US norm | vs within category |
| Type of Ig treatment |  |  |  | NS |  |  | NS |
| SCIG | 424 | 46.9 (10.7) | <0.05 |  | 41.3 (12.0) | <0.05 |  |
| IVIG | 519 | 45.6 (10.9) | <0.05 |  | 40.6 (12.3) | <0.05 |  |
| Perception of disease control |  |  |  |  |  |  |  |
| Well/completely | 518 | 48.0 (10.3) | <0.05 |  | 45.2 (11.5) | <0.05 |  |
| Adequately | 331 | 44.7 (10.9) | <0.05 | <0.05^a^ | 37.1 (11.0) | <0.05 | <0.05^a^ |
| Less than adequately/poorly | 75 | 41.5 (11.1) | <0.05 |  | 30.8 (9.2) | <0.05 |  |
| Experiences fatigue or low energy^b^ |  |  |  |  |  |  |  |
| Always | 356 | 42.0 (11.3) | <0.05 | <0.05 | 35.1 (11.0) | <0.05 | <0.05 |
| Occasionally | 368 | 47.1 (9.8) | <0.05 |  | 42.6 (11.5) | <0.05 |  |
| Never | 211 | 51.6 (8.7) | <0.05 |  | 47.7 (10.7) | <0.05 |  |
| Ig Treatment location |  |  |  | NS |  |  | <0.05^c^ |
| SCIG: home—self | 405 | 47.0 (10.8) | <0.05 |  | 41.6 (11.8) | <0.05 |  |
| IVIG: home—nurse/self | 210 | 45.5 (10.3) | <0.05 |  | 41.8 (12.7) | <0.05 |  |
| IVIG: infusion suite | 147 | 45.9 (10.8) | <0.05 |  | 39.2 (11.6) | <0.05 |  |
| IVIG: hospital out-patient | 69 | 47.1 (11.9) | <0.05 |  | 42.0 (13.0) | <0.05 |  |
| IVIG: Other | 69 | 44 (11.9) | <0.05 |  | 42.0 (12.2) | <0.05 |  |
| Bother of Ig treatment |  |  |  |  |  |  |  |
| Not at all | 287 | 49.9 (9.9) | NS |  | 42.4 (12.3) | <0.05 |  |
| A little bit | 439 | 45.9 (10.4) | <0.05 | <0.05^d,e,f,g,h,I,j,k^ | 41.3 (12.1) | <0.05 | <0.05^e,f,g^ |
| Moderately | 158 | 42.8 (10.9) | <0.05 |  | 38.4 (11.1) | <0.05 |  |
| Quite a bit/extremely | 52 | 38.7 (12.1) | <0.05 |  | 38.3 (13.4) | <0.05 |  |

^a^ Comparisons between “well-to-adequately controlled” and “poorly controlled”

^b^ Does the patient experience periods of fatigue or low energy between Ig treatments—Comparisons between “always” and “occasionally”, “always” and “never”, and “occasionally” and “never”

^c^ Comparisons between IVIG: Home—nurse/self and IVIG: Infusion suite

^d^ Comparisons between “not at all” and “a little bit” bothered

^e^ Comparisons between “not at all” and “moderately” bothered

^f^ Comparisons between “not at all” and “quite a bit/extremely” bothered

^g^ Comparisons between “a little bit” and “moderately” bothered

^h^ Comparisons between “a little bit ” and “quite a bit/ extremely” bothered

^I^ Comparisons between “moderately” and “quite a bit/ extremely” bothered

^j^ Comparisons between “moderately” and “a little bit” bothered

^k^ Comparisons between “quite a bit/extremely” and “moderately” bothered

*Ig* immunoglobulin, *IVIG* intravenously administered immunoglobulin, *QOL* quality of life, *SCIG* subcutaneously administered immunoglobulin, *SD* standard deviation, *NS* not significant
