# Supplementary material for: Health-Related Quality of Life in Adult Patients with Common Variable Immunodeficiency Disorders and Impact of Treatment
Source: J Clin Immunol. 2017 May 23;37(5):461–75. doi: 10.1007/s10875-017-0404-8 (PMC5489588; doi:10.1007/s10875-017-0404-8)
Supplement: Supplementary file 21 — (DOCX 47 kb). [file 10875_2017_404_MOESM14_ESM.docx]

**Supplemental Data**

**Health Status in Adult Patients with Common Variable Immunodeficiency and the Impact of Treatment**

*Journal of Clinical Immunology*

Nicholas L. Rider · Carleigh Kutac · Joud Hajjar · Chris Scalchunes · Filiz O. Seeborg · Marcia Boyle · Jordan S. Orange

**Correspondence:** Dr. Nicholas L. Rider, D.O., Section of Immunology, Allergy and Rheumatology, Texas Children’s Hospital, 1102 Bates St, Suite 330, Houston, TX, 77030, USA
E-mail: nlrider@bcm.edu

**Table S3** Univariate analysis: variables affecting the Physical Component Scores for patients with CVID (unadjusted model)

| PCS category | PCS (overall mean 40.9 [95% CI: 40.1, 41.7]) | | | | |
| --- | --- | --- | --- | --- | --- |
|  | PCS comparator | | | Difference from mean PCS | |
|  | Comparator | Mean PCS (95% CI) | *P*-value | Difference (95% CI) | *P*-value |
| Overall bother when getting Ig therapy | Not bothered at all | 42.4 (41.0, 43.8) | <0.05 |  |  |
| Bothered a little bit |  |  |  | -1.1 (-2.9, 0.7) | NS |
| Moderately bothered |  |  |  | -4.0 (-6.4, -1.6) | <0.05 |
| Bothered quite a bit to extremely bothered |  |  |  | -4.1 (-7.7, -0.5) | <0.05 |
| Missing |  |  |  | -11.2 (-19.2, -3.2) | <0.05 |
| Fatigue or low energy (wear-off) between treatments | Always | 35.1 (33.9, 36.3) | <0.05 |  |  |
| Occasionally |  |  |  | 7.5 (5.9, 9.1) | <0.05 |
| Never |  |  |  | 12.6 (10.7, 14.5) | <0.05 |
| Missing |  |  |  | 5.0 (-2.0, 12.0) | NS |
| Permanent impairments | 0–2 | 42.6 (41.8, 43.4) | <0.05 |  |  |
| ≥3 |  |  |  | -4.1 (-4.8, -3.4) | <0.05 |
| Permanent digestive and/or lung impairment | No | 43.9 (42.9, 44.8) | <0.05 |  |  |
| Yes |  |  |  | -7.6 (-9.17, -6.11) | <0.05 |
| Gender | Male | 54.5 (51.1, 57.8) | <0.05 |  |  |
| Female |  |  |  | -7.6 (-9.4, -5.8) | <0.05 |
| Age, years | Age 45–54 years | 42.0 (40.4, 43.6) | <0.05 |  |  |
| 18–24 |  |  |  | 7.9 (4.0, 11.7) | <0.05 |
| 25–34 |  |  |  | 3.3 (0.3, 6.4) | <0.05 |
| 35–44 |  |  |  | -0.1 (-2.8, 2.5) | NS |
| 55–64 |  |  |  | -3.8 (-6.0, -1.7) | <0.05 |
| 65–74 |  |  |  | -2.7 (-5.1, -0.2) | <0.05 |
| ≥75 |  |  |  | -4.3 (-8.2, -0.4) | <0.05 |
| Limitations in past 12 months | No limitations | 53.3 (52.0, 54.7) | <0.05 |  |  |
| Slight |  |  |  | -7.5 (-9.1, -5.8) | <0.05 |
| Moderate |  |  |  | -18.8 (-20.5, -17.1) | <0.05 |
| Severe |  |  |  | -24.9 (-26.6, -22.9) | <0.05 |
| Missing |  |  |  | -22.0 (-26.2, -17.9) | <0.05 |
| Health status in past  12 months | Good | 44.4 (43.4, 45.4) | <0.05 |  |  |
| Excellent |  |  |  | 13.1 (9.9, 16.4) | <0.05 |
| Very Good |  |  |  | 6.5 (4.9, 8.0) | <0.05 |
| Fair |  |  |  | -10.3 (-11.8, -8.9) | <0.05 |
| Poor |  |  |  | -16.5 (-18.5, 14.5) | <0.05 |
| Very Poor |  |  |  | -21.1 (-25.0, -17.1) | <0.05 |
| Missing/no response |  |  |  | -9.2 (-12.7, -5.7) | <0.01 |
| Treatment location | Home | 41.6 (40.6, 42.6) | <0.05 |  |  |
| Hosptial outpatient |  |  |  | -3.29 (-6.3, -0.3) | <0.05 |
| Infusion suite |  |  |  | -2.49 (-4.7, -0.3) | <0.05 |
| Other |  |  |  | 0.38 [-2.7, 3.4) | NS |
| Missing |  |  |  | -3.0 (-2.7, 3.4) | NS |
| Treating doctor | Allergist/immunologist | 41.6 (40.7, 42.4) | <0.05 |  |  |
| Other |  |  |  | -4.8 (-7.0, -2.5) | <0.05 |
| How well Ig controls PIDD | Completely to well controlled | 45.2 (44.2, 46.2) | <0.05 |  |  |
| Adequately controlled |  |  |  | -8.2 (-9.7, -6.6) | <0.05 |
| Less than adequately to poorly controlled |  |  |  | -14.4 (-17, -11.7) | <0.05 |
| Missing |  |  |  | -13.7 (-18.8, -8.6) | <0.05 |
| Hospitalized overnight in past 12 months | No | 42.7 (41.9, 43.6) | <0.05 |  |  |
| Yes |  |  |  | -7.7 (-9.6, -5.9) | <0.05 |
| Hospitalized in ICU in past 12 months | No | 41.3 (40.5, 42.1) | <0.05 |  |  |
| Yes |  |  |  | -8.4 (-13.3, -3.5) | <0.05 |
| Inpatient operations in past 12 months | No | 42.4 (41.4, 43.3) | <0.05 |  |  |
| Yes |  |  |  | -6.6 (-9.1, -4.1) | <0.05 |
| Outatient operations in past 12 months | No | 42.3 (41.4, 43.3) | <0.05 |  |  |
| Yes |  |  |  | -4.0 (-5.9, -2.0) | <0.05 |
| Serious infections | No | 45.0 (42.8, 47.2) | <0.05 |  |  |
| Yes |  |  |  | -4.7 (-7.1. -2.3) | <0.05 |
| Severe side effects | No | 43.2 (42.3, 44.1) | <0.05 |  |  |
| Yes |  |  |  | -7.8 (-9.5. -6.2) | <0.05 |
| Missing |  |  |  | -4.3 (-8.8, 0.3) | NS |

*CI* confidence interval, *CVID* common variable immunodeficiency, *ICU* intensive care unit, *Ig* immunoglobulin, *NS* not significant, *PCS* Physical Component Score, *PIDD* primary immunodeficiency diseases
