# Supplementary material for: Health-Related Quality of Life in Adult Patients with Common Variable Immunodeficiency Disorders and Impact of Treatment
Source: J Clin Immunol. 2017 May 23;37(5):461–75. doi: 10.1007/s10875-017-0404-8 (PMC5489588; doi:10.1007/s10875-017-0404-8)
Supplement: Supplementary file 22 — (DOCX 43 kb). [file 10875_2017_404_MOESM15_ESM.docx]

**Supplemental Data**

**Health Status in Adult Patients with Common Variable Immunodeficiency and the Impact of Treatment**

*Journal of Clinical Immunology*

Nicholas L. Rider · Carleigh Kutac · Joud Hajjar · Chris Scalchunes · Filiz O. Seeborg · Marcia Boyle · Jordan S. Orange

**Correspondence:** Dr. Nicholas L. Rider, D.O., Section of Immunology, Allergy and Rheumatology, Texas Children’s Hospital, 1102 Bates St, Suite 330, Houston, TX, 77030, USA
E-mail: nlrider@bcm.edu

**Table S4** Univariate analysis: variables affecting the Mental Component Scores for patients with CVID (unadjusted model)

| MCS category | MCS (overall mean 46.9 [95% CI: 45.5; 46.9]) | | | | |
| --- | --- | --- | --- | --- | --- |
|  | MCS comparator | | | Difference from mean MCS | |
|  | Comparator | Mean MCS  (95% CI) | *P*-value | Difference (95% CI) | *P*-value |
| Overall bother when getting Ig therapy | Not bothered at all | 49.9 (48.7, 51.1) | <0.05 |  |  |
| Bothered a little bit |  |  |  | -4.0 (-4.2, -1.3) | <0.05 |
| Moderately bothered |  |  |  | -7.1 (-6.1, -2.2) | <0.05 |
| Bothered quite a bit to extremely bothered |  |  |  | -11.2 (-9.9, -4.0) | <0.05 |
| Missing |  |  |  | -5.7 (-8.7, 6.5) | NS |
| Fatigue or low energy  (wear-off) between treatments | Always | 42.0 (41.0, 43.1) | <0.05 |  |  |
| Occasionally |  |  |  | 5.1 (1.3, 4.2) | <0.05 |
| Never |  |  |  | 9.6 (3.6, 7.3) | <0.05 |
| Missing |  |  |  | 2.1 (-7.5, 5.2) | NS |
| Permanent impairments | 0–2 | 46.9 (46.1, 47.6) | <0.05 |  |  |
| ≥3 |  |  |  | -4.6 (-6.6, -2.7) | <0.05 |
| Permanent digestive and/or lung impairment | No | 47.1 (46.3, 48.0) | <0.05 |  |  |
| Yes |  |  |  | -2.4 (-3.8, -1.0) | <0.05 |
| Age, years | Age 45–54 years | 43.6 (42.2, 45.0) | <0.05 |  |  |
| 18–24 |  |  |  | 4.0 (-2.7, 3.6) | <0.05 |
| 25–34 |  |  |  | 3.1 (-1.1, 3.8) | <0.05 |
| 35–44 |  |  |  | 2.7 (0.1, 4.3) | <0.05 |
| 55–64 |  |  |  | 1.8 (0.4, 3.9) | NS |
| 65–74 |  |  |  | 5.6 (2.8, 6.7) | <0.05 |
| ≥75 |  |  |  | 6.6 (1.7, 8.0) | <0.05 |
| Limitations in past 12 months | No limitations | 52.5 (51.0, 54.1) | <0.05 |  |  |
| Slight |  |  |  | -4.5 (-6.4, -2.6) | <0.05 |
| Moderate |  |  |  | -8.9 (-10.8, -6.9) | <0.05 |
| Severe |  |  |  | -12.4 (-14.7, -10.1) | <0.05 |
| Missing |  |  |  | -10.8 (-15.6, -6.0) | <0.05 |
| Health status in past  12 months | Good | 46.6 (45.4, 47.7) | <0.05 |  |  |
| Excellent |  |  |  | 7.0 (3.2, 10.7) | <0.05 |
| Very Good |  |  |  | 4.9 (3.0, 6.7) | <0.05 |
| Fair |  |  |  | -2.8 (-4.4, -1.1) | <0.05 |
| Poor |  |  |  | -5.7 (-8.0, -3.3) | <0.05 |
| Very Poor |  |  |  | -9.4 (-14.0, -4.8) | <0.05 |
| Missing/no response |  |  |  | -2.9 (-7.0, 1.2) | NS |
| How well Ig controls PIDD | Completely to well controlled | 48.0 (47.1, 48.9) | <0.05 |  |  |
| Adequately controlled |  |  |  | -3.4 (-4.9, -1.9) | <0.05 |
| Less than adequately to poorly controlled |  |  |  | -6.6 (-9.1, -4.0) | <0.05 |
| Missing |  |  |  | -4.8 (-9.7, 0.1) | NS |
| Severe side effects | No | 47.6 (46.8, 48.5) | <0.05 |  |  |
| Yes |  |  |  | -5.3 (-6.8, -3.7) | <0.05 |
| Missing |  |  |  | -0.1 (-4.1, 4.0) | NS |

*CI* confidence interval, *CVID* common variable immunodeficiency, *Ig* immunoglobulin, *MCS* Mental Component Score, *NS* not significant, *PIDD* primary immunodeficiency diseases
